# Supplementary material for: Intention to use maternity waiting home and associated factors among pregnant women in Gamo Gofa zone, Southern Ethiopia, 2019
Source: PLoS One. 2021 May 13;16(5):e0251196. doi: 10.1371/journal.pone.0251196 (PMC8118329; doi:10.1371/journal.pone.0251196)
Supplement: S1 Fig — (DOCX) [file pone.0251196.s001.docx]

**
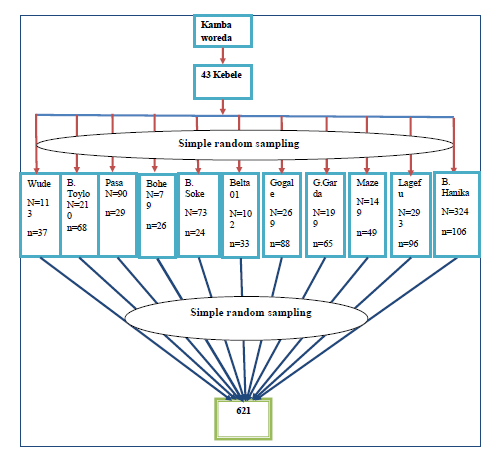
**

**S1 Fig. Diagrammatic presentation of sampling procedure used to recruit pregnant women in Kamba District, Gamo Gofa Zone, Southern Ethiopia, 2019.**
