# Supplementary material for: Intention to use maternity waiting home and associated factors among pregnant women in Gamo Gofa zone, Southern Ethiopia, 2019
Source: PLoS One. 2021 May 13;16(5):e0251196. doi: 10.1371/journal.pone.0251196 (PMC8118329; doi:10.1371/journal.pone.0251196)
Supplement: S1 Table — (DOCX) [file pone.0251196.s003.docx]

**S1 Table. English version questionnaire.**

| **Part 1. Socio demographic related questions** | | | | | | | **Skip pattern** | |
| --- | --- | --- | --- | --- | --- | --- | --- | --- |
| S. no | | Questions | Response options | | | |  | |
| 1 | | How old are you? | ________ years old (age in years) | | | |  | |
| 2 | | What religion are you following? | 1. Orthodox 2. Muslim 3. Protestant 4. Others(specify)_______ | | | |  | |
| 3 | | What is your educational status? | 1. Unable to read and write 2. Only read and write 3. Primary education (grade 1-8) 4. Secondary education and above | | | |  | |
| 4 | | What is your marital status? | 1. Married 2. Single 3. Widowed 4. Divorced | | | |  | |
| 5 | | What is your occupation? | 1. House wife 2. Merchant 3. Government employee 4. Others (specify) __________ | | | |  | |
| 6 | | What is your ethnicity? | 1. Gamo 2. Gofa 3. Others (specify) __________ | | | |  | |
| 7 | | How much is your monthly income? | _____________Ethiopian birr | | | |  | |
| **Part II. Obstetrics related questions** | | | | | | |  | |
| 8 | How many pregnancies do you have experienced? | | ______________(in number) | | | |  | |
| 9 | Have you ever give birth before? | | 1. Yes 2. No | | | | If no, skip to Q12 | |
| 10 | How many times did you give birth before? | | __________(in number) | | | |  | |
| 11 | Where did you attend your child birth/s? | | 1. At home 2. At health institution | | | |  | |
| 12 | Do you have ANC follow up for current pregnancy? | | 1. Yes 2. No | | | | If no, skip to Q14 | |
| 13 | If yes to question No.12, How many times did you attended ANC follow up in the current pregnancy? | | 1. Once 2. Two times 3. Three times 4. Four times and above | | | |  | |
| **Part III. Attitude measurement**  **3.1. Direct attitude measurement** | | | | | | | | |
| 14 | 1. For me staying in health center for institutional delivery 15 days before giving birth is___________. | | 1. Bad---1---2---3---4---5--- Good 2. Useless---1---2---3---4---5--- Useful 3. Unpleasant---1---2---3---4---5--- Pleasant 4. Boring---1---2---3---4---5--- Interesting | | | | |  |
| **3.2. Indirect attitude measurement**  **A. Behavioral beliefs measurement** | | | | | | | | |
| 15 | **1.** Staying in health center for institutional delivery 15 days before giving birth will help me to get delivery by health professionals & prevent myself from death related to delivery. | | | | | 1. Strongly disagree 2. Disagree 3. Neutral 4. Agree 5. Strongly agree | |  |
| 16 | **2.** Staying in health center for institutional delivery 15 days before giving birth will help me to get healthy child. | | | | | 1. Strongly disagree 2. Disagree 3. Neutral 4. Agree 5. Strongly agree | |  |
| 17 | **3.** Staying in health center for institutional delivery 15 days before giving birth will help be to be happy & reduce fear of labor. | | | | | 1. Strongly disagree 2. Disagree 3. Neutral 4. Agree 5. Strongly agree | |  |
| 18 | **4.** Staying in health center for institutional delivery 15 days before giving birth will help me to get better ANC services. | | | | | 1. Strongly disagree 2. Disagree 3. Neutral 4. Agree 5. Strongly agree | |  |
| 19 | **5.** Staying in health center for institutional delivery 15 days before giving birth will help me to get better health information on child immunization, family planning & personal hygiene. | | | | | 1. Strongly disagree 2. Disagree 3. Neutral 4. Agree 5. Strongly agree | |  |
| **B. Evaluation of outcomes** | | | | | | | | |
| 20 | For me getting delivery by health professionals & prevent myself from death related to delivery is___. | | | | | 1. Very Bad 2. Bad 3. Neutral 4. Good 5. Very good | |  |
| 21 | For me getting healthy child is______. | | | | | 1. Very Bad 2. Bad 3. Neutral 4. Good 5. Very good | |  |
| 22 | For me being happy & reduce fear of labor is____. | | | | | 1. Very Bad 2. Bad 3. Neutral 4. Good 5. Very good | |  |
| 23 | For me getting better ANC services is____. | | | | | 1. Very Bad 2. Bad 3. Neutral 4. Good 5. Very good | |  |
| 24 | For me getting health information on immunization, family planning & other health service is_______. | | | | | 1. Very Bad 2. Bad 3. Neutral 4. Good 5. Very good | |  |
| **3.3. Subjective norm measurement**  **Direct subjective norm measurement** | | | | | | | | |
| 25 | Most people who are important to me will approve of my staying in health center for institutional delivery 15 days before giving birth. | | | | | 1. Strongly disagree 2. Disagree 3. Neutral 4. Agree 5. Strongly agree | |  |
| 26 | Most people who are important to me will think that I should stay in health center for institutional delivery 15 days before giving birth. | | | | | 1. Strongly disagree 2. Disagree 3. Neutral 4. Agree 5. Strongly agree | |  |
| 27 | Most people who like me want my staying in health center for institutional delivery 15 days before giving birth. | | | | | 1. Strongly disagree 2. Disagree 3. Neutral 4. Agree 5. Strongly agree | |  |
| 28 | It is expected of me that I have to stay in health center for institutional delivery 15 days before giving birth. | | | | | 1. Strongly disagree 2. 2.Disagree 3. Neutral 4. Agree 5. Strongly agree | |  |
| **Indirect subjective norm measurement**  **A. Normative beliefs** | | | | | | | | |
| 29 | My mother thinks that I should stay in health center for institutional delivery 15 days before giving birth. | | | | | 1. Strongly disagree 2. Disagree 3. Neutral 4. Agree 5. Strongly agree | |  |
| 30 | My husband thinks that I should stay in health center for institutional delivery 15 days before giving birth. | | | | | 1. Strongly disagree 2. Disagree 3. Neutral 4. Agree 5. Strongly agree | |  |
| 31 | My neighbor thinks that I should stay in health center for institutional delivery 15 days before giving birth. | | | | | 1. Strongly disagree 2. Disagree 3. Neutral 4. Agree 5. Strongly agree | |  |
| 32 | Health extension workers approve my staying in health center for institutional delivery 15 days before giving birth. | | | | | 1. Strongly disagree 2. Disagree 3. Neutral 4. Agree 5. Strongly agree | |  |
| **B. Motivation to comply** | | | | | | | | |
| 33 | My mother’s approval of staying in health center for institutional delivery 15 days before giving birth is important to me. | | | | 1. Not very much 2. Not Much 3. Neutral 4. Much 5. Very much | | |  |
| 34 | My husband’s approval of staying in health center for institutional delivery 15 days before giving birth is important to me. | | | | 1. Not very much 2. Not Much 3. Neutral 4. Much 5. Very much | | |  |
| 35 | My neighbors’ approval of staying in health center for institutional delivery 15 days before giving birth is important to me. | | | | 1. Not very much 2. Not Much 3. Neutral 4. Much 5. Very much | | |  |
| 36 | Health extension worker’s approval of staying in health center for institutional delivery 15 days before giving birth is important to me. | | | | 1. Not very much 2. Not Much 3. Neutral 4. Much 5. Very much | | |  |
| **Part IV. Perceived behavioral control measurement**  **4.1 Direct perceived behavioral control measurement** | | | | | | | | |
| 37 | For me staying in health center for institutional delivery 15 days before giving birth is_________. | | | 1. Difficult ----1---2---3---4---5---Easy 2. Not under my control---1---2---3---4---5---Under my control 3. Sudden ----1---2---3---4---5---Planned 4. Conditional ---1---2---3---4---5---Unconditional | | | |  |
| **4.2 Indirect perceived behavioral control measurement**  **Control beliefs measurement** | | | | | | | | |
| 38 | When my gestational age advances, I cannot get transportation/ walk long distance/ to go & stay in health center for institutional delivery prior to 15 days before giving birth. | | | | | 1. Very Unlikely 2. Unlikely 3. Neutral 4. Likely 5. Very likely | |  |
| 39 | If I stay in health center I could not get enough food to stay in health center for institutional delivery 15 days before giving birth. | | | | | 1. Very Unlikely 2. Unlikely 3. Neutral 4. Likely 5. Very likely | |  |
| 40 | If my gestation increases I cannot get individuals to take to health center for staying in it for institutional delivery 15 days before giving birth. | | | | | 1. Very Unlikely 2. Unlikely 3. Neutral 4. Likely 5. Very likely | |  |
| 41 | Difficult to get individuals that can give care for my family once I left to maternity waiting home | | | | | 1. Very Unlikely 2. Unlikely 3. Neutral 4. Likely 5. Very likely | |  |
| **Power of control measurement** | | | | | | | | |
| 42 | Lack of transportation /long distance/ makes it difficult for me to go & stay in health center for institutional delivery prior to 15 days before giving birth. | | | | | 1. Strongly disagree 2. Disagree 3. Neutral 4. Agree 5. Strongly agree | |  |
| 43 | Food insecurity in health center makes it difficult for me to stay in health center for institutional delivery 15 days before giving birth. | | | | | 1. Strongly disagree 2. Disagree 3. Neutral 4. Agree 5. Strongly agree | |  |
| 44 | Hard ship of staying/desolate surrounding makes it more difficult for me to stay in health center for institutional delivery 15 days before giving birth. | | | | | 1. Strongly disagree 2. Disagree 3. Neutral 4. Agree 5. Strongly agree | |  |
| 45 | Difficulty to get individuals that can give care for my family makes it difficult for me to stay in health center for institutional delivery prior to 15 days before giving birth. | | | | | 1. Strongly disagree 2. Disagree 3. Neutral 4. 4.Agree 5. Strongly agree | |  |
| **Intention measurements** | | | | | | | | |
| 46 | I am intended to stay in health center for institutional delivery 15 days before giving birth | | | | | 1. Strongly disagree 2. Disagree 3. Neutral 4. Agree 5. Strongly agree | |  |
| 47 | I will stay in health center for institutional delivery 15 days before giving birth. | | | | | 1. Strongly disagree 2. Disagree 3. Neutral 4. Agree 5. Strongly agree | |  |
| 48 | I want to stay in health center for institutional delivery 15 days before giving birth. | | | | | 1. Strongly disagree 2. Disagree 3. Neutral 4. Agree 5. Strongly agree | |  |
| 49 | I like to stay in health center for institutional delivery 15 days before giving birth. | | | | | 1. Strongly disagree 2. Disagree 3. Neutral 4. Agree 5. Strongly agree | |  |
| **Past experiences** | | | | | | | | |
| 50 | Have you ever been used maternity waiting home before? | | | | | 1. Yes 2. No | |  |
|  |  |  |  |  |  |  |  |  |
| 51 | What is your reason to use MWH? | | | | | 1. Fear of labor illness 2. To get enough rest and free from workload 3. To get better health care from health professionals 4. Fear of death related to delivery 5. To get healthy child 6. Others (specify) _____ | |  |
| 51 | How many days have you stayed in MWH? | | | | | 1. Only 15 days 2. Less than 15 days 3. More than 15 days | |  |

**Thank you for your participation!**
