# Supplementary material for: Intention to use maternity waiting home and associated factors among pregnant women in Gamo Gofa zone, Southern Ethiopia, 2019
Source: PLoS One. 2021 May 13;16(5):e0251196. doi: 10.1371/journal.pone.0251196 (PMC8118329; doi:10.1371/journal.pone.0251196)
Supplement: S2 Table — (DOCX) [file pone.0251196.s004.docx]

**S2 Table. Amharic version questionnaire (በአማርኛ የተዘጋጀ መጠይቅ).**

| **ተ.ቁ** | **መጠይቆች** | | **የመልስ አማራጮች** | ወደ--- ይለፉ | |
| --- | --- | --- | --- | --- | --- |
| **ክፍል 1፡- ማህበራዊ እና ስነህዝባዊ ጥያቄዎችን በተመለከተ** | | | | | |
| 1 | አሁን እድሜዎ ስንት ነዉ? | | ---------------ዓመት (በዓመት) |  | |
| 2 | የምን ሃይማኖት ተከታይ ነዎት? | | 1. ኦርቶዶክስ 2. እስልምና 3. ፕሮትስታንት 4. ሌላ(ይገለፅ)---------- |  | |
| 3 | ብሄርዎ ምን ነዉ? | | 1. ጋሞ 2. ጎፋ 3. ሌላ(ይገለፅ)------ |  | |
| 4 | የትምህርት ደረጃዎ እስከ ምን ድረስ ነዉ? | | 1. ማንበብና መጻፍ አልችልም 2. ማንበብና መጻፍ ብቻ እችላለሁ 3. የመጀመሪያ ደረጃ ት/ት አተጠናቅቂያለሁ 4. የሁለተኛ ደረጃ ት/ት እና ከዚያ በላይ ተምሪያለሁ |  | |
| 5 | የጋብቻ ሁኔታዎ ምንድን ነዉ? | | 1. ያገባች 2. ያላገባች 3. ባሉዋ የሞተባት 4. አግብታ የፈታች |  | |
| 6 | ስራዎ ምንድን ነዉ? | | 1. የቤት እመቤት 2. ነጋደ 3. የመንግስት ሰራተኛ 4. ሌላ (ይገለፅ)------ |  | |
| **7** | በወር ምንያህል ገቢ ያገኛሉ? | | ------------------ የኢትዮጵያ ብር |  | |
| **ከእርግዝና እና ወሊድ ጋር የተያያዙ ጥያቀዎች** | | | |  | |
| 8 | ያሁኑ ስነትኛ እርግዝናዎ ነዉ? | | ---------------(በቁጥር) |  | |
| 9 | ከዚህ በፊት ልጅ ወልደዉ ያዉቃሉ? | | 1. አዎ ወልጀ አዉቃለሁ |  | |
|  |  |  | 1. አይ ወልጀ አላዉቅም | መልስዎአይ ወልጀ አላዉቅም ከሆን ወደ ጥያቂ 14 ይሂዱ | |
| 10 | የት ነዉ የወለዱት? | | 1. እቤት 2. በጤና ተቁዋም |  | |
| 11 | ስነት ጊዚ ዎልደዋል? | | -**-----------** (በቁጥር) |  | |
| 12 | በዚህ እርግዚናዎ የእርግዝና ክትትል አድርገዋል? | | 1. አዎ 2. አይ |  | |
| 13 | ስንት ጊዜ ክትትል አድርገዋል? | | 1. አንድ ጊዜ  2. ሁሌት ጊዜ  3. ሶስት ጊዜ  4. አራት ጊዜ እና ከዚያ በላይ |  | |
| **ክፍል 3፡- የአመለካከት መለኪያ ጥያቀዎች**  **3.1 ቀጥተኛ የአመለካከት መለኪያዎች** | | | | | |
| 14 | 1. ለኔ ከወሊድ በፊት ጤና ጣቢያ ላይ 15 ቀን መቀመጥ--------------፡፡ | | 1. መጥፎ ---1---2---3---4---5---ጥሩ ነዉ 2. ጥቅም አልባ----1---2----3---4---5---ጠቃሚ ነዉ 3. አስቀያሚ ----1---2----3---4---5--- አስደሳች 4. አሰልቺ---1---2.----3---4---5--- ማራኪ | |  |
| 1. **2. በተጉዋዳኝ አመለካከትን የሚለኩ ጥያቀዎች**   **ሀ. የአድራጎት ሁኔታ ያለዉን እምነት መለኪያዎች** | | | | | |
| 15 | 1. በጤና ተቁዋም ለመዉለድ ከወሊድ በፊት ጤና ጣቢያ ላይ 15 ቀን መቆየት የባለሙያ እርዳታ እንዳገኝና ከወሊድ ጋር ተያይዞ ከሚመጣ ሞት እራሲን ለመከላከል ይረዳኛል፡፡ | | 1. በጣም አልስማማም 2. አልስማማም 3. አይመለከተኝም 4. እስማማለሁ 5. በጣም እስማማለሁ |  | |
| 16 | 2. በጤና ተቁዋም ለመዉለድ ከወሊድ በፊት ጤና ጣቢያ ላይ 15 ቀን መቆየት ጤናማ ልጅ እንዳገኝ ይረዳኛል፡፡ | | 1. በጣም አልስማማም 2. አልስማማም 3. አይመለከተኝም 4. እስማማለሁ 5. በጣም እስማማለሁ |  | |
| 17 | 1. .በጤና ተቁዋም ለመዉለድ ከወሊድ በፊት ጤና ጣቢያ ላይ 15 ቀን መቆየት የምጥን ፍርሀት ለመቀነስ እና ደስተኛ እንድሆን ይረዳኛል፡፡ | | 1. በጣም አልስማማም 2. አልስማማም 3. አይመለከተኝም 4. እስማማለሁ 5. በጣም እስማማለሁ |  | |
| 18 | 1. በጤና ተቁዋም ለመዉለድ ከወሊድ በፊት ጤና ጣቢያ ላይ 15 ቀን መቆየት መቆየት የተሻለ የርገዝና ክትትል እንዳገኝ ይረዳኛል፡፡ | | 1. በጣም አልስማማም 2. አልስማማም 3. አይመለከተኝም 4. እስማማለሁ 5. በጣም እስማማለሁ |  | |
| 19 | 1. በጤና ተቁዋም ለመዉለድ ከወሊድ በፊት ጤና ጣቢያ ላይ 15 ቀን መቆየት የተሻለ ስለልጄ ክትባት ፤ ስለቤተስብ ምጣኔ አገልግሎት እና ስለ ግል ንጽህናዬ የጤና መረጃ እንዳገኝ ይረዳኛል፡፡ | | 1. በጣምአልስማማም 2. አልስማማም 3. አይመለከተኝም. 4. እስማማለዉ 5. በጣም እስማማለዉ |  | |
| **ለ. ዉጤቱን ስለማመዘን በተመለከተ (Evaluation of outcomes)** | | | | | |
| 20 | ለኔ በጤና ባለሙያ መዉለድና እራስን ከወሊድ ጋር ተያይዞ ከሚመጣ ሞት መከላከል----------፡፡ | | 1. በጣም መጥፎ ነዉ 2. መጥፎ ነዉ 3. አይመለከተኝም 4. ጥሩ ነዉ 5. በጣም ጥሩ ነዉ |  | |
| 21 | ለኔ ጤናማ ልጅ መዉለድ----------፡፡ | | 1. በጣም መጥፎ ነዉ 2. መጥፎ ነዉ 3. አይመለከተኝም 4. ጥሩ ነዉ 5. በጣም ጥሩ ነዉ |  | |
| 22 | ለኔ የምጥን ፍርሀት መቀነስ እና ደስተኛ መሆን--------------፡፡ | | 1. በጣም መጥፎ ነዉ 2. መጥፎ ነዉ 3. አይመለከተኝም 4. ጥሩ ነዉ 5. በጣም ጥሩ ነዉ |  | |
| 23 | ለኔ የተሻለ የእርግዝና ክትትል ማግኘት-----------------፡፡ | | 1. በጣም መጥፎ ነዉ 2. መጥፎ ነዉ 3. አይመለከተኝም 4. ጥሩ ነዉ 5. በጣም ጥሩ ነዉ |  | |
| 24 | ለኔ የተሻለ ስለ ልጄ ክትባት፤ስለቤተስብ ምጣኔ አገልግሎት እና ስለ ግል ንጽህናዬ የጤና መረጃ ማግኘት-------------፡፡ | | 1. በጣም መጥፎ ነዉ 2. መጥፎ ነዉ 3. አይመለከተኝም 4. ጥሩ ነዉ 5. በጣም ጥሩ ነዉ |  | |
| **የይሉኝታ መለክያ ጥያቄዎች**  **ቀጥተኛ የይሉኝታ መለክያዎች** | | | | | |
| **25** | ለኔ የቅርቤ እምላቸዉ ብዙ ሰዎች ከወሊድ በፊት ጤና ጣቢያ ላይ በጤና ተቁዋም ለመዉለድ 15 ቀን እንድቆይ ይወስናሉ፡፡ | | 1. በጣም አልስማማም 2. አልስማማም 3. አይመለከተኝም 4. እስማማለሁ 5. በጣም እስማማለሁ |  | |
| **26** | ለኔ የቅርቤ እምላቸዉ ብዙ ሰዎች እኔ ከወሊድ በፊት ጤና ጣቢያ ላይ በጤና ተቁዋም ለመዉለደረ 15 ቀን እንድቆይ ያስባሉ፡፡ | | 1. በጣም አልስማማም 2. አልስማማም 3. አይመለከተኝም 4. እስማማለሁ 5. በጣም እስማማለሁ |  | |
| **27** | እኔን የሚወዱኝ ብዙ ሰዎች እኔ ከወሊድ በፊት ጤና ጣቢያ ላይ በጤና ተቁዋም ለመዉለድ 15 ቀን እንድቆ ይይፈልጋሉ፡፡ | | 1. በጣም አልስማማም 2. አልስማማም 3. አይመለከተኝም 4. እስማማለሁ 5. በጣምእስማማለሁ |  | |
| **28** | በጤና ተቁዋም ለመዉለድ ከወሊድ በፊት ጤና ጣቢያ ላይ 15 ቀን መቆየት ከኔ ይጠበቃል፡፡ | | 1. በጣም አልስማማም 2. አልስማማም 3. አይመለከተኝም 4. እስማማለሁ 5. በጣም እስማማለሁ |  | |
| **በተዘዋዋሪ ይሉኝታን የሚለኩ ጥያቀዎች**  **ሀ. ልማዳዊ እምነት** | | | | | |
| **29** | እናቴ እኔ ከወሊድ በፊት ጤና ጣቢያ ላይ በጤና ተቁዋም ለመዉለድ 15 ቀን መቆየት እንዳለብኝ ታስባለች፡፡ | | 1. በጣም አልስማማም 2. አልስማማም 3. አይመለከተኝም 4. እስማማለሁ 5. በጣም እስማማለሁ |  | |
| **30** | ባለቤቴ እኔ ከወሊድ በፊት ጤና ጣቢያ ላይ በጤና ተቁዋም ለመዉለድ 15 ቀን መቆየት እንዳለብኝ ያስባል፡፡ | | 1. በጣም አልስማማም 2. አልስማማም 3. አይመለከተኝም 4. እስማማለሁ 5. በጣም እስማማለሁ |  | |
| **31** | ጎሮበቴ እኔ ከወሊድ በፊት ጤና ጣቢያ ላይ በጤና ተቁዋም ለመዉለድ 15 ቀን መቆየት እንዳለብኝ ያስባል/ታስባለች፡፡ | | 1. በጣም አልስማማም 2. አልስማማም 3. አይመለከተኝም. 4. እስማማለሁ 5. በጣም እስማማለሁ |  | |
| **32** | ጤና ኤክስቴንሽን ሰራተኞችን እኔ በጤና ተቁዋም ለመዉለድ ከወሊድ በፊት ጤና ጣቢያ ላይ 15 ቀን መቆየት እንዳለብኝ ያስባሉ/ታስባለች፡፡ | | 1. በጣም አልስማማም 2. አልስማማም 3. አይመለከተኝም. 4. እስማማለሁ 5. በጣም እስማማለሁ |  | |
| **ለ. ለማድረግ የሚያገፋፉ ምክንያቶች** | | | | | |
| **33** | እናቴ እኔ በጤና ተቁዋም ለመዉለድ ከወሊድ በፊት ጤና ጣቢያ ላይ 15 ቀን መቆየት እንዳለብኝ መወሰንዋ ለኔ-------፡፡ | | 1. በጣም ብዙም አይደለም 2. ብዙም አይደለም 3. አይታወቅም 4. ብዙ ነዉ 5. በጣም ብዙ ነዉ |  | |
| **34** | ባለቤቴ እኔ በጤና ተቁዋም ለመዉለድ ከወሊድ በፊት ጤና ጣቢያ ላይ 15 ቀን መቆየት እንዳለብኝ መወሰኑ ለኔ------፡፡ | | 1. በጣም ብዙም አይደለም 2. ብዙም አይደለም 3. አይታወቅም 4. ብዙ ነዉ 5. በጣም ብዙ ነዉ |  | |
| 35 | ጎሮቤቴ እኔ በጤና ተቁዋም ለመዉለድ ከወሊድ በፊት ጤና ጣቢያ ላይ 15 ቀን መቆየት እንዳለብኝ መወሰኑዋ ለኔ-----፡፡ | | 1. በጣም ብዙም አይደለም 2. ብዙምአ ይደለም 3. አይታወቅም 4. ብዙ ነዉ 5. በጣም ብዙ ነዉ |  | |
| **36** | ጤና ኤክስቴንሽን ሰራተኞችን እኔ ከወሊድ በፊት ጤና ጣቢያ ላይ በጤና ተቁዋም ለመዉለድ 15 ቀን መቆየት እንዳለብኝ የወሰኑት/ችዉ ለኔ----------፡፡ | | 1. በጣም ብዙም አይደለም 2. ብዙም አይደለም 3. አይታወቅም 4. ብዙ ነዉ 5. በጣም ብዙነዉ |  | |
| **የተገመተ የባህሪ ቁጥጥር መለኪያ ጥያቄዎች**  **ቀጥተኛ የተገመተ ባህሪይ ቁጥጥር መለኪያ ጥያቄዎች** | | | | | |
| **37** | ለኔ ከወሊድ በፊት ጤና ጣቢያ ላይ በጤና ተቁዋም ለመዉለድ 15 ቀን መቆየት-----፡፡ | | 1. ከባድ ነዉ...1...2...3...4...5...ቀላል 2. ከቁጥጥሬ ዉጪ ነዉ...1...2...3... 4...5...በኔ ቁጥጥር ነዉ 3. ድንገተኛ ነዉ...1...2....3...4...5... የታቀደ ነዉ 4. እንደሁኔታዉ ነዉ...1...2.... 3 ... 4...5...ያለገደብነዉ |  | |
| **በተዘዋዋሪ የተገመተ የባህሪ ቁጥጥር መለኪያ ጥያቄዎች**  **የመቆጣጠር እምነትቶች መለኪያ ጥያቄዎች** | | | | | |
| **38** | እርግዝናዬ እያደገ ስሄድ ከወሊድ በፊት ጤና ጣቢያ ላይ በጤና ተቁዋም ለመዉለድ 15 ቀን ለመቆየት ትራንስፖርት ላላገኝ/ ረጅም ርቀት ላልሄድ እችላለሁ፡፡ | 1. በጣም ሊሆን የማይችል ነዉ 2. ሊሆን የማይችል ነዉ 3. አይታወቅም 4. ሊሆን የሚችል ነዉ 5. በጣም ልሆን የሚችል ነዉ | |  | |
| **39** | ከወሊድ በፊት 15 ቀን ጤና ጣቢያ ላይ የምቆይ ከሆነ በቂ ምግብ ላላገኝ እችላለሁ፡፡ | 1. በጣም ሊሆን የማይችል ነዉ 2. ሊሆን የማይችል ነዉ 3. አይታወቅም 4. ሊሆን የሚችል ነዉ 5. በጣም ልሆን የሚችል ነዉ | |  | |
| **40** | ጽንሴ እያደገ ሲሄድ በጤና ተቁዋም ለመዉለድ ከወሊድ በፊት 15 ቀን እንድቆይ ወደ ጤና ጣቢያ የሚወስደኝን ስዉ ላጣ እችላለዉ፡፡ | 1. በጣም ሊሆን የማይችል ነዉ 2. ሊሆን የማይችል ነዉ 3. አይታወቅም 4. ሊሆን የሚችል ነዉ 5. በጣም ሊሆን የሚችል ነዉ | |  | |
| **41** | ወደ እናቶች ማቆያ ከሄድኩኝ በኃላ ቤተሰቤን የሚንከባክብ ሰዉ ማግኘት ሊከብደኝ ይችላል፡፡ | 1. በጣም ሊሆን የማይችል ነዉ 2. ሊሆን የማይችል ነዉ 3. አይታወቅም 4. ሊሆን የሚችል ነዉ 5. በጣም ሊሆን የሚችል ነዉ | |  | |
| **የመቆጣጠር አቅም መለኪያ ጥያቄዎች** | | | | | |
| **42** | ትራንስፖርት ማጣት/ረጅም ርቀት ከወሊድ በፊት በጤና ተቁዋም ለመዉለድ ጤና ጣቢያ ላይ 15 ቀን ለመቆየት እንዳልሄድና እንዳልቆይ ያደርጋሉ፡፡ | 1. በጣም አልስማማም 2. አልስማማም 3. አይመለከተኝም 4. እስማማለሁ 5. በጣም እስማማለሁ | |  | |
| **43** | በጤና ጣቢያዎች የምግብ ዋስትና አለመኖር ከወሊድ በፊት በጤና ተቁዋም ለመዉለድ ጤና ጣቢያ ላይ 15 ቀን ለመቆየት እንዳልሄድና እንዳልቆይ ያደርጋል፡፡ | 1. በጣም አልስማማም 2. አልስማማም 3. አይመለከተኝም 4. እስማማለሁ 5. በጣም እስማማለሁ | |  | |
| **44** | ቦታዉ ለመቆየት ክባድ/ብቸኝነት ያጠቃዉ መሆኑ ከወሊድ በፊት በጤና ተቁዋም ለመዉለድ ጤና ጣቢያ ላይ 15 ቀን እንዳልቆይ ያደርኛል፡፡ | 1. በጣም አልስማማም 2. አልስማማም 3. አይመለከተኝም 4. እስማማለሁ 5. በጣም እስማማለሁ | |  | |
| **45** | ወደ እናቶች ማቆያ ከሄድኩኝ በኃላ ቤተሰቤን የሚንከባክብ ሰዉ ባለመኖሩ ምክንያት በጤና ተቁዋም ለመዉለድ ከወሊድ በፊት ጤና ጣቢያ ላይ 15 ቀን መቆየት ሊከብደኝ ይችላል፡፡ | 1. በጣም አልስማማም 2. አልስማማም 3. አይመለከተኝም 4. እስማማለዉ 5. በጣም እስማማለዉ | |  | |
| **የፊላጎት መለክያ ጥያቄዎች** | | | | | |
| **46** | በጤና ተቁዋም ለመዉለድ ከወሊድ በፊት ጤና ጣቢያ ላይ 15 ቀን የመቆየት ሀሳብ አለኝ፡፡ | 1. በጣም አልስማማም 2. አልስማማም 3. አይመለከተኝም 4. እስማማለዉ 5. በጣም እስማማለዉ | |  | |
| **47** | ከወሊድ በፊት በጤና ተቁዋም ለመዉልድ ጤና ጣቢያ ላይ 15 ቀን እቆያለዉ፡፡ | 1. በጣም አልስማማም 2. አልስማማም 3. አይመለከተኝም 4. እስማማለሁ 5. በጣምእስማማለሁ | |  | |
| **48** | በጤና ተቁዋም ለመዉለድ ከወሊድ በፊት ጤና ጣቢያ ላይ 15 ቀን መቆየት እፈልጋለዉ፡፡ | 1. በጣም አልስማማም 2. አልስማማ 3. አይመለከተኝም 4. እስማማለሁ 5. በጣም እስማማለሁ | |  | |
| **49** | ከወሊ ድበፊት በጤና ተቁዋም ለመዉለድ ጤና ጣቢያ ላይ 15 ቀን መቆየት እወዳለዉ፡፡ | 1. በጣም አልስማማም 2. አልስማማም 3. አይመለከተኝም 4. እስማማለሁ 5. በጣም እስማማለሁ | |  | |
| **ልምድን በተመለከተ** | | | | | |
| **50** | ከዚህ በፊት የእናቶች ማቆያን ተጠቅመዉ ያቃሉ | 1. አዎ | |  | |
|  |  | 2. ተጠቀሚ አላዉቅም | |  | |
| **51** | በምን ምከንያት ነዉ የተጠቀሙት | 1. የምጥ ህመም ፍራቻ 2. ከስራ ጫና ለመገላገል እና በቂ እረፍ ት ለማገኘት 3. የተሻለ የጤና አገልግሎት ከጤና ባለሙያ ለማግኘት 4. ከወሊድ ጋር ተያይዞ ከሚመጣ ሞት ፍራቻ 5. ጤናማ ልጅ ለማግኘት 6. ሌላ (ይገለፅ)............................ | |  | |
| **52** | በእናቶች ማቆያ ላይ ስንት ቀን ቆይተዋል | 1. 15 ቀን ብቻ 2. ከ15 ቀን በታች 3. ከ15 ቀን በላይ | |  | |

**ስለተሳትፎዎ እናመሰግናለን!!**
