# Supplementary material for: Intention to use maternity waiting home and associated factors among pregnant women in Gamo Gofa zone, Southern Ethiopia, 2019
Source: PLoS One. 2021 May 13;16(5):e0251196. doi: 10.1371/journal.pone.0251196 (PMC8118329; doi:10.1371/journal.pone.0251196)
Supplement: S3 Table — (DOCX) [file pone.0251196.s005.docx]

**S3 Table. Gamogna version questionnaire (Gamothon giigida oyshata)**

| **Dereteththanne dereteththagisha xeeliya oyshata** | | | | | |  | | |
| --- | --- | --- | --- | --- | --- | --- | --- | --- |
| **Paydo** | **Oyshata** | **Zaaro doorota** | | | | **Aadha** | | |
| 1 | Ha7i laythay aappunne? | ________ Laythay(laythan) | | | |  | | |
| 2 | Aza ammano kaaleeti? | 1. Orttodokisse 2. Isilaame 3. Pirotestante 4. Harata(qoncissa) _____ | | | |  | | |
| 3 | Oonateththay | 1. Gamo 2. Gofa 3. Harata (qoncissa)_____ | | | |  | | |
| 4 | Timirtte deththay? | 1. Nabbabone xaafo dandduwa 2. Nabbabo xaafo xalala dandda7ayis 3. Koyro detha timirte wursadis 4. Nam7anththo detha timirtenne hessappe | | | |  | | |
| 5 | Gelo eketetha hanotay? | 1. Gelidaro 2. Gelentaro 3. Azinay hayqqidaro 4. Sayo __________ | | | |  | | |
| 6 | Aysethiya ooso hanotay aaze? | 1. Keetha aayo 2. Zal77ancho 3. Kawo oosancho 4. Harata(qoncissa)_____ | | | |  | | |
| 7 | Aginan ayi keena gabe demmeeti? | _____________(Tophiyaa Biran) | | | |  | | |
| **Wodeththa-attethara oykketida oyshata** | | | | | |  | | |
| 8 | Hayssiaappuntho wodetha ateththee? | ______________ (paydon) | | | |  | | |
| 9 | Hayssape kase na7a yelideti? | 1. Ee 2. Akkay | | | | Akkay gidiko Oysha12k | | |
| 10 | Hayssape kase aappun toho na7a yelideti? | __________(paydon) | | | |  | | |
| 11 | Awan yelidetii (yelo bessa)? | 1. Deetha sohon 2. Payyatetha naagisiza sohon | | | |  | | |
| 12 | Ha7i wodeththa ateththi kaaleteththi ooththetii? | 1. Ee 2. Akkay | | | | Akkay gidiko Oysha14k | | |
| 13 | Aappun wode kaaleteththi ooththideeti? | 1. Issi wode 2. Ubba wode 3. Hedzdzu wode 4. Oyiddu wode | | | |  | | |
| **Gaatha Xeela geedaro oyshata; suure xeelo geedaro** | | | | | | | | |
| 14 | Taas na7a yelanaappe kasetada payateththa naagiso eqotan 15 gallassi uteththay____. | 1. Iita 1----2----3----4-----5----lo7oko 2. Go7ay bawa 1----2---3----4----5---go7ees 3. Lo7ontayisa----1----2----3----4----5----uppayissizayissa 4. Salethizayssa------1 ----2---3---4—5 | | | | |  | |
| **Hara ogera xeela geedariza oyshsata polo hanotan diza ammano geedazata** | | | | | | | | |
| 15 | Payateythanaagiso keetha go77etanaw yelanappe sinthe payatetha xaaban 15 gallas gam77oy erranchcho maado demmanaw-nne yelora gakkidi oykettidi yiza hayqqoppe bana teqqanas maaddees. | | | | 1. Daroppe ekkike 2. Ekkike 3. Wochchenna 4. Ekkadis 5. Daroppe ekkadis | |  | |
| 16 | Payateytha naagiso keetha go77etanaw yelanappe sinthe payatetha xaaban 15 gallas gam77oy paya na7a demmanaw maadees. | | | | 1. Daroppe ekkike 2. Ekkike 3. Wochchenna 4. Ekkadis 5. Daroppe ekkadis | |  | |
| 17 | **Payyatetha keethan haggazettanas yelanappe sinthe payyatetha xaaban 15 gallas gam7oy yelo miixa babo shakannasinne upayssan daana mala tna maadees.** | | | | 1. Daroppe ekkike 2. Ekkike 3. Wochchenna 4. Ekkadis 5. Daroppe ekkadis | |  | |
| 18 | **Payetetha keethan yelanas yeloppe sinthe payateththa xxaban 15 gallassi gam77oy lo7o wodeththa kaaleteththi demmana mala tana maaddees.** | | | | 1. Daroppe ekkike 2. Ekkike 3. Wochchenna 4. Ekkadis 5. Daroppe ekkadis | |  | |
| 19 | **Payetetha keethan yelanas yeloppe sinthe payateththa xxaban 15 gallassi gam77oy lo7o wodeththa kaaleteththi demmana mala tana maaddees.** | | | | 1. Daroppe ekkike 2. Ekkike 3. Wochchenna 4. Ekkadis 5. Daroppe ekkadis | |  | |
| **Muruttaa geeddaro xeelo** | | | | | | | | |
| 20 | **Taas payateththa hillanchchan yeloy-nne banateththa yelora gaththin yiza hayqqooppe teqqeththay_____.** | | | | 1. **Daroiita** 2. **Iita** 3. **Wochchenna** 4. **Lo7o** 5. **Daroppe lo7o** | |  | |
| 21 | Taas paya naa yeloy ______. | | | | 1. **Daroiita** 2. **Iita** 3. **Wochchenna** 4. **Lo7o** 5. **Daroppe lo7o** | |  | |
| 22 | Taas yelo-miixa babo guuthoynne upa7ettizassa gidoy __. | | | | 1. **Daroiita** 2. **Iita** 3. **Wochchenna** 4. **Lo7o** 5. **Daroppe lo7o** | |  | |
| 23 | Taas lo7o wodeththan-nne kaaletethi demoy____. | | | | 1. **Daroiita** 2. **Iita** 3. **Wochchenna** 4. **Lo7o** 5. **Daroppe lo7o** | |  | |
| 24 | Taas lo7o tana kittibaate, soo asa geeddaro haggazo-nne ta geeshateththa payateththa markkateththa demoy _____. | | | | 1. **Daroiita** 2. **Iita** 3. **Wochchenna** 4. **Lo7o** 5. **Daroppe lo7o** | |  | |
| **Woygandde geeddaro oyshshata; suure woyggandde geeddaro** | | | | | | | | |
| 25 | **Daroppe taas mata getettiza assati yelanappe sinththe payateththa xaaban 15 gallassi payateththa keeththan yelanaw 15 gallassi gam77ana –mala qachchoosona.** | | | | 1. Daroppe ekkike 2. Ekkike 3. Wochchenna 4. Ekkadis 5. Daroppe ekkadis | |  | |
| 26 | **Daroppe taas mata ta giza assati tani yelana-ppe sinththe payateththa keeththan yelanaw 15 gallassi gam77ana mala qoppoosona.** | | | | 1. Daroppe ekkike 2. Ekkike 3. Wochchenna 4. Ekkadis 5. Daroppe ekkadis | |  | |
| 27 | **Daroppe taas mata ta giza assati tani yelana-ppe sinththe payateththa keeththan yelanaw 15 gallassi gam77ana mala koyosona.** | | | | 1. Daroppe ekkike 2. Ekkike 3. Wochchenna 4. Ekkadis 5. Daroppe ekkadis | |  | |
| 28 | **Payyatetha keethan yelanas yelope sinthe payyatetha xaaban15 gallasi gam7ana mala taappe naagetees.** | | | | 1. Daroppe ekkike 2. Ekkike 3. Wochchenna 4. Ekkadis 5. Daroppe ekkadis | |  | |
| **Citas dumma ogera woyggande geedaro oyshata**  **Lose ammano** | | | | | | | | |
| 29 | **Ta aaya tani yeloppe sinththe payyatetha xaaban payyateththa keeththan yellanaw 15 gallassi gam7ana mala qoppawus** | | | | 1. Daroppe ekkike 2. Ekkike 3. Wochchenna 4. Ekkadis 5. Daroppe ekkadis | |  | |
| 30 | **Ta keeththa aaway tani yeloppe sinththe payyatetha xaaban payyateththa keeththan yellanaw 15 gallassi gam7ana mala qoppees** | | | | 1. Daroppe ekkike 2. Ekkike 3. Wochchenna 4. Ekkadis 5. Daroppe ekkadis | |  | |
| 31 | **Ta shooroy tani yeloppe sinththe payyatetha xaaban payyateththa keeththan yellanaw 15 gallassi gam7ana mala qoppawus.** | | | | 1. Daroppe ekkike 2. Ekkike 3. Wochchenna 4. Ekkadis 5. Daroppe ekkadis | |  | |
| 32 | **Payyateththa dalggiso osanchati tani yeloppe sinththe payyatetha xaaban payyateththa keeththan yellanaw 15 gallassi gam7ana mala qopposonna/qoppawus.** | | | | 1. Daroppe ekkike 2. Ekkike 3. Wochchenna 4. Ekkadis 5. Daroppe ekkadis | |  | |
| **Oothanas denththithiza gaasota** | | | | | | | | |
| 33 | **Ta aayiya tani yeloppe sinththe payyatetha xaaban payyateththa keeththan yellanaw 15 gallassi gam7ana mala qachidayssi taas____.** | | | 1. **Keehippe daro gidenna** 2. **Daro gidenna** 3. **Eretena** 4. **Daro** 5. **Keehi daro** | | |  | |
| 34 | **Ta keeththa aaway tani yeloppe sinththe payyatetha xaaban payyateththa keeththan yellanaw 15 gallassi gam7ana mala qachidayssi taas___________.** | | | 1. **Keehippe daro gidenna** 2. **Daro gidenna** 3. **Eretena** 4. **Daro** 5. **Keehi daro** | | |  | |
| 35 | **Ta shooroy tani yeloppe sinththe payyatetha xaaban payyateththa keeththan yellanaw 15 gallassi gam7ana mala qachidayssi taas_____.** | | | 1. **Keehippe daro gidenna** 2. **Daro gidenna** 3. **Eretena** 4. **Daro** 5. **Keehi daro** | | |  | |
| 36 | Payyateththa dalggiso osanchati tani yeloppe sinththe payyatetha xaaba payyateththa keeththan yellanaw 15 gallassi gam7ana mala qachidayssi/ra taas______. | | | 1. **Keehippe daro gidenna** 2. **Daro gidenna** 3. **Eretena** 4. **Daro** 5. **Keehi daro** | | |  | |
| **Heezettida kanddo kaaleteththa geedara**  **Suure heezettida kanddo kaaleteththa geedara** | | | | | | | | |
| 37 | **Taas yeloppe sinththe payyatetha xaaban payyateththa keethan yelanaw 15 gallassi gam7o_____________.** | | 1. **Deexo ----1------2-----3-----4----5----kawushe** 2. **Ta kaaletethappe kare—1---2---3---4---5---ta kaaletethana** 3. **Qoppontta---1-----2----3-----4-----5----halchetidayssa** 4. **Hano mala----1----2----3----4-----5---zawi** | | | |  | |
| **Dumma ogera heezettida kanddo kaaleteththa geedara**  **Kaaletetha geedara** | | | | | | | | |
| 38 | **Ta yedeththay dicci bishin yelanape sinthe payyateththa xaaban payyateththa keethan yelanas 15 gallassa gam7anaw toga demmannaw/haaho buussi beetontayssa gidana.** | | | | 1. **Daroppe ekkike** 2. **Ekkike** 3. **Wochchenna** 4. **Ekkadis** 5. **Daroppe ekkadis** | |  | |
| 39 | **Yellope sinthe 15 gallassa payyateththa xaaban gam7iza gidikko giddiza kaththi demonta agana.** | | | | 1. **Daroppe ekkike** 2. **Ekkike** 3. **Wochchenna** 4. **Ekkadis** 5. **Daroppe ekkadis** | |  | |
| 40 | **Attethay gujishin payyatetha keethan yellanaw yelloppe sinthe 15 gallassa gam7anaw payyatetha xaaba epiza asi dhayandes.** | | | | 1. **Daroppe ekkike** 2. **Ekkike** 3. **Wochchenna** 4. **Ekkadis** 5. **Daroppe ekkadis** | |  | |
| 41 | **Yelanaw aayeta gam7osso bidappe guyyen ta soo asa xeeliza ase demmoy dexxana damndda7ees.** | | | | 1. **Daroppe ekkike** 2. **Ekkike** 3. **Wochchenna** 4. **Ekkadis** 5. **Daroppe ekkadis** | |  | |
| **Kaaleteththa wolqqa geedaro** | | | | | | | | |
| 42 | Tiranssiportte dhayoy/haahotethi yelanape sinthe payyateththa keethan yelanaw payyateththa xaaban 15 gallas gam7anaw baanasinne gam7ontta mala ooththandosona**.** | | | | 1. **Daroppe ekkike** 2. **Ekkike** 3. **Wochchenna** 4. **Ekkadis** 5. **Daroppe ekkadis** | |  | |
| 43 | **Payyateththa xaabatan diza kaththa demo dhaysi yeloppe sinththe payateththa keeththan yelanaw payyateththa xaaban 15 gallas gam7anaw bonttanne gam7ontta mala ooththanaw dandda7oosona.** | | | | 1. **Daroppe ekkike** 2. **Ekkike** 3. **Wochchenna** 4. **Ekkadis** 5. **Daroppe ekkadis** | |  | |
| 44 | **Bessay gam7annaw deexo woykko bizzatethi qohidayoissa gidikko yeloppe sinththe payyateththa keeththan yelanaw payyateththa xaaban 15 gallas gam7ontta mala ooththandees.** | | | | 1. **Daroppe ekkike** 2. **Ekkike** 3. **Wochchenna** 4. **Ekkadis** 5. **Daroppe ekkadis** | |  | |
| 45 | **Aayeta yeloso bidappe guyyen soo asa xeeliza asa dhayissa gaasson payyateththa keeththan yelanaw yeloppe sinththe payyateththa xaaban 15 gallassa gam7oy taas deexana** | | | | 1. **Daroppe ekkike** 2. **Ekkike** 3. **Wochchenna** 4. **Ekkadis** 5. **Daroppe ekkadis** | |  | |
| **Gaaththa qoppa geedaro** | | | | | | | | |
| 46 | **Payyatetha keethan yelanaw yeloppe sinththe payyateththa xaaban 15 gallassi gam7o qoppay dees.** | | | | 1. **Daroppe ekkike** 2. **Ekkike** 3. **Wochchenna** 4. **Ekkadis** 5. **Daroppe ekkadis** | |  | |
| 47 | **Payyatetha keethan yelanaw yeloppe sinththe payyateththa xaaban 15 gallassi gam7anna.** | | | | 1. **Daroppe ekkike** 2. **Ekkike** 3. **Wochchenna** 4. **Ekkadis** 5. **Daroppe ekkadis** | |  | |
| 48 | **Payyatetha keethan yelanaw yeloppe sinththe payyateththa xaaban 15 gallassi gam7anas koyais.** | | | | 1. **Daroppe ekkike** 2. **Ekkike** 3. **Wochchenna** 4. **Ekkadis** 5. **Daroppe ekkadis** | |  | |
| 49 | **Payyatetha keethan yelanaw yeloppe sinththe payyateththa xaaban 15 gallassi gam7o dossayis** | | | | 1. **Daroppe ekkike** 2. **Ekkike** 3. **Wochchenna** 4. **Ekkadis** 5. **Daroppe ekkadis** | |  | |
| **Go7etetha lose xeelo** | | | | | | | | |
| 50 | **Hayssappe sinththe aayetas yelanaw payyateththa xaaban gam7izayssa go7eti ereetii?** | | | | 1. **Ee** 2. **Akkay** | | |  |
| 51 | **Aaza gaason go77ettidettii?** | | | | 1. **Miixa sako babos** 2. **Ooththo deexoppe attanas** 3. **Lo7o payyateththa hagazo hiilanchata demmananas** 4. **Yelora gathin yiza hayqqo babos** 5. **Payya na7a demmanaw** 6. **Haratakka(qoncissa)** _____ | | |  |
| 52 | **Aayeti yelanaw gam7izza bessan aappun gallassi gam7idetii?** | | | | - 1. **15 gallassi xalla**   2. **15 gallassappe garsse**   3. **15 gallassappe bolla** | | |  |

Qoppa gishetetha gishas galatays!!
