# Supplementary material for: Intention to use maternity waiting home and associated factors among pregnant women in Gamo Gofa zone, Southern Ethiopia, 2019
Source: PLoS One. 2021 May 13;16(5):e0251196. doi: 10.1371/journal.pone.0251196 (PMC8118329; doi:10.1371/journal.pone.0251196)
Supplement: S1 File — (DOCX) [file pone.0251196.s006.docx]

**S1 File. English version consent form.**

Hello my name is _________________ (name of data collector). I am____________________ (the data collector briefly introduce him/herself), and I am here to collect data on “Intention to use maternity waiting home and associated factors among pregnant women” for research purpose. The objective of this study is to assess intention to use maternity waiting home and associated factors among pregnant women in Gamo Gofa zone. The benefit of your participation in this study is to improve maternal and neonatal health in your community as well as a country as a whole by increasing institutional delivery. Hence, your trustworthy and frank participation is ultimately important to achieve this goal. All the information that you provide must be kept confidentially, and your name and information will not be disclosed. The information you give is only disclosed to the investigators and they will use it only for this research purposes. You’ve a full right to not respond to all or part of the questions.

Are you voluntary to participate in this study? 1. Yes 2. No

**Thank you!!**

**Name of data collector: ____________________**

**Cell phone of data collector: _______________________**

**Date of data collection:** _________/_________/
