# Supplementary material for: Intention to use maternity waiting home and associated factors among pregnant women in Gamo Gofa zone, Southern Ethiopia, 2019
Source: PLoS One. 2021 May 13;16(5):e0251196. doi: 10.1371/journal.pone.0251196 (PMC8118329; doi:10.1371/journal.pone.0251196)
Supplement: S2 File — (DOCX) [file pone.0251196.s007.docx]

**S2 File. Amharic version consent form (በአማረኛ የተዘጋጀ የተሳታፊወች ስምምነት ቅጽ).**

ጤና ይስጥልኝ! ስሜ------------ይባላል(የመረጃ ሰብሳቢዉ ስም)፡፡ እኔ----------------------(መረጃ ሰብሳቢዉ/ዋ እራሱን/ስዋን ባናጭሩ ያስተዋዉቃል/ታስተዋዉቃለች). እናም ከዚህ የተገኘሁት**“የእናቶችን ማቆያ የመጠቀም ፍላጎትና ተዛማች ችግሮች በጋሞ ጎፋ ዞን በሚገኙ ነፍሰ ጡር እናቶች”** በምል ርዕስ ጥናት መረጃ ለመሰብሰብ ነዉ፡፡ የዚህ ጥናት አላማ የእናቶችን ማቆያ የመጠቀም ፍላጎትና ተዛማች ችግሮችን በጋሞ ጎፋ ዞን በሚገኙ ነፍሰ ጡር እናቶች ለመዳሰስ ነው፡፡ በዚህ ጥናት ውስጥ ያለዎት ተሳትፎ በማህበረሰብዎ ውስጥ የእናቶች እና የጨቅላ ህጻናት ጤንነትን ለማሻሻልና እንደዚሁም በጤና ተቁዋም የመዉለድ ልምድን እነዲሁም በአጠቃላይ በሀገር አቀፍ ደረጃ ለማስፋፋት ይጠቅማል፡፡ ስለሆነም ይህንን ግብ ለማሳካት እርስዎ በታማኝነት እና ከልብ በመሳተፍ የሚሰጡን መረጃ እጅግ ጠቃሚና የላቀ ነው፡፡ ስለዚህ ለመጠይቆችን በተቻለዎት መጠን በትክክልና በጥንቃቄ ምላሽ እንዲሰጡኝ በትህትና እጠይቃለሁ፡፡ የሚሰጡን መረጃ ሁሉ በሚስጥር የሚያዝ ሲሆን ስምና ማንነትዎ ይፋ አይደረግም፡፡ ስለ እርስዎ መረጃ የሚያውቁት አጥኚዎች ብቻ ሲሆኑ መረጃውም ለጥናቱ አላማ ብቻ የሚያውል ይሆናል፡፡ ጥያቄዎቹን በሙሉም ሆነ በከፊል ያለመመለስ ሙሉ መብት አለዎት፡፡

ለመሳተፍ ፍቃደኛ ነዎት? 1. አዎ 2. አይደለሁም

እናመሰግናለን!!

**የመረጃ ሰብሳቢዉ ስም፡ ------------------**

**የመረጃ ሰብሳቢዉ ስልክ ቁጥር፡ ----------------------**

**መረጃ የተሰበሰበበት ቀን፡ -------/----------------/**
