# Supplementary material for: Intention to use maternity waiting home and associated factors among pregnant women in Gamo Gofa zone, Southern Ethiopia, 2019
Source: PLoS One. 2021 May 13;16(5):e0251196. doi: 10.1371/journal.pone.0251196 (PMC8118329; doi:10.1371/journal.pone.0251196)
Supplement: S3 File — (DOCX) [file pone.0251196.s008.docx]

**S3 File. Gamogna Version Consent Form (Gamothon giigida qopa gishetha issippetetha qixxe)**

Saroy gido! Ta sunthay ----------- geetettays (qopa shiishizayssa suntha).Tani--------- (qopa shiishizayssi banatetha qanthara qoncisees) gidishin hayssan tani beetizay **“Aayeta payatetha naagisoheeran gam7o go7etethas koshshanne gaasotizametota Gaamo Gofa zoonen Kambba woradan de7iza shaaraa maccasata bolla”**giza kaaraan gaththi xeelo oothashe qopa shiishanasa.

Ha gathi-xeelos Dane qopay ayeeta payatetha naagiso heeran gam7os go7a dassanne; gaasotida metota Gaamo Gofa zone Kambba woraddan de7iza shaara maccasata hanota xeelossa.

Ha xeelo gaathan hintes gishetetha go7ay heera giddon aayeetanne-yelaga nayta payayetetha giigissanassinne payatetha naagiso keetha go7etetha lose ubba dere guppantho dethan dalggissanas maaddees. Hessa gishas hintte immiza qopay ubbay wozanthan oykkettizayssa gidishe sunthay-nne oonateththay oonassika qoncce oosettenna, hintte immiza markkateththa erizay gaththi-xeelizayissa kanchche gidishin markkateththaykka gaththi xeelo qopas kanchche peshizayissa gidees. Oyshatappe kumethaza woykko zawattidayssatas zaaro imo ago kumeththa maatay de7ees.

Issipetanas koyeeti? 1. Ee 2. Akkayi

Galatayis!

**Qopa shiishiza bitane suntha: ________________**

**Qopa shiishiza bitane silkke paydo: ____________**

**Qopay shiiqida gallasa**: ____/____/_____
