# Supplementary material for: Intention to use maternity waiting home and associated factors among pregnant women in Gamo Gofa zone, Southern Ethiopia, 2019
Source: PLoS One. 2021 May 13;16(5):e0251196. doi: 10.1371/journal.pone.0251196 (PMC8118329; doi:10.1371/journal.pone.0251196)
Supplement: Attachment — Submitted filename: Reviewer 1.docx [file pone.0251196.s010.docx]

**Intention to use maternity waiting home and associated factors among pregnant women in Kamba District, Gamo Gofa zone, Southern Ethiopia, 2019**

**Yibelu Bazezaw ^1*^, Wubishet Gezimu^2,^ Mekuanint Taddele^3^, Tewodros Eshete^3^**

*Corresponding author

^1^ Department of Midwifery, College of Health sciences, Debre Markos University, Debre Markos, Ethiopia

^2^ Department of Nursing, College of Health sciences, Mettu University, Mettu, Ethiopia

^3^ Department of Public health, College of Health sciences, Debre Markos University, Debre Markos, Ethiopia

**Authors address**

YB- [yibebazezew5106@gmail.com](mailto:yibebazezew5106@gmail.com)

WG- [wubishet151@gmail.com](mailto:wubishet151@gmail.com)

MT- [mekutaddele@gmail.com](mailto:mekutaddele@gmail.com)

TE- [teddyeshet143@gmail.com](mailto:teddyeshet143@gmail.com)

**Intention to use maternity waiting home and associated factors among pregnant women in Kamba District, Gamo Gofa zone, Southern Ethiopia, 2019**

**ABSTRACT**

**Background:** A maternity waiting home is a temporary residence in which pregnant women from remote areas wait for their childbirth. It is an approach targeted to advance access to emergency obstetric care services especially in hard to reach areas to escalate institutional delivery to reduce complications that occur during childbirth. Apart from the availability of this service, the intention of pregnant women to utilize the existing service is very important to achieve its goals.

Research aim: This study aimed to assess the intention to use maternity waiting homes and associated factors among pregnant women.

**Methods:** Community-based cross-sectional study was conducted among 621 pregnant women using a multistage sampling technique from March 10- April 10 /2019 by using structured questionnaires through face to face interviews. The collected data was entered into Epi-Data version 3.1 and analyzed using SPSS version 24 statistical package. Logistic regression analysis was used to test the association. All variables at p-value < 0.25 in bivariate analysis were entered into multivariate analysis. Lastly, a significant association was declared at a P-value of < 0.05 with 95% CI.

**Results:** In this study, the intention to use maternity waiting home was 295(48.76% 995%CI: 47%-55%)). Occupation (government employee) (AOR:2.87,95%CI: 1.54-5.36), previous child birth history (AOR:2.1,95%CI:1.22-3.57), past experience in maternity waiting home use AOR:4.35,95%CI:2.63-7.18), direct (AOR:1.57,95%CI:1.01-2.47) and indirect (AOR: 2.18, 1.38,3.44) subject norms and direct (AOR:3.00,95%CI:2.03-4.43), and indirect (1.84,95%CI:1.25-2.71) perceived behavioral control of respondents were significantly associated variables with intention to use maternity waiting home.

**Conclusion and Recommendations:** Since the magnitude of intention to use maternity waiting home in this study area was unsatisfactory and occupation, subjective norm, and perceived behavioral control were identified factors affecting intention, intervention programs, such as health education, strengthening and integration of community in health system programs need to be provided.

**Keywords:** Intention, Maternity Waiting Home, Pregnant women, and Kamba district

**Background**

World health organization (WHO) defined Maternity Waiting Home (MWH) as a temporary housing service nearby to health facilities, in which pregnant women wait for childbirth [1].

Endorsing MWH near hospitals is a strategy that can reduce inequity, through improving poorer women’s access to facilities that enables to provide advanced management of childbirth complications, and highly profitable, and cheap approach to decrease maternal morbidity and mortality as well as a low-cost solution to access skilled birth attendant in remote areas [1-4]. It is also a life-changing innovative approach, which was established as one of the three institutional innovations that have substantially contributed to progress in the strengthening of health facilities and persons [5]. Different literature in Africa also indicated the importance of MWH, and the challenges to utilize it [6- 9].

Maternal Waiting Home is important to educate the women, counseling about pregnancy, childbirth, and care of the newborn infant and family planning, and also it offers the opportunity for women to come closer to the clinic before labour starts, avoiding the reluctance to walk several distances after labour begins. It increases institutional deliveries and accordingly decreases maternal mortality caused by the delays in reaching obstetric care [3, 10].

Due to difficulty in transportation to and from the hospital, and the high number of obstetric emergencies in the area, the first MWH in Ethiopia was built in 1976 [11]. The use of Maternity Waiting Home for rural populations with limited access to Emergency Obstetric Care (EmOC) is one of the policy options to overcome the low utilization of skilled birth attendants [12]. Currently in Ethiopia, the Amhara region is the top in MWHs/rooms coverage with 72% followed by SNNPR (57%) and Oromo region (56%); least (8%) in the Gambella region [13].

Maternal Waiting Home is the only feasible option that is currently open for remote societies in which pregnant women can be moved near to essential and emergency obstetric care services centers before the commencement of labor when she is strong enough to do so and while the roads are open [14].

The health-seeking behavior of the mothers, inadequate progressive planning for delivery; the women’s perception of service benefits (attitude); women’s perception about social pressure from important others (normative beliefs); the previous history of obstetric complications, and the availability of MWH at health institutions are factors that are related to the will of the women to utilize MWH [15, 16]. However, there has been little attempt made to find out the intentions of mothers to use MWH services in our country.

Globally, an estimated total of 10.7 million women died in a year between 1990 and 2015 due to maternal causes. This catastrophic event in developing regions is coarsely 20 folds higher than that of developed regions. Approximately 99% of the estimated global maternal deaths occurred in developing regions in 2015. Of this sub-Saharan Africa alone accounted for roughly 66% of maternal deaths followed by Southern Asia [17]. According to the United Nations sustainable development goal (SDG) three target one plan, the global maternal mortality ratio would be less than 70 per 100,000 live births by 2030 and thus each country in the world will be required to reduce MMR by at least 7.5% each year between 2016 and 2030 to achieve this target[18].

Access to comprehensive emergency obstetric care is limited in Ethiopia; and due to lack of modern transport, people use a locally made stretcher to carry laboring mother to the health facility by community members, the most commonly used means of transportation for cases necessitating referral [11]. In Ethiopia, the maternal mortality ratio still remains high, which is 412 per 100,000 live births. According to EDHS 2016 survey report, institutional delivery is only 26%. Though, escalating institutional deliveries is vital to reduce maternal and neonatal mortality. Home delivery is still common, due to distance, inaccessibility, and lack of appropriate facilities, lack of access to health facilities in rural areas; primarily in hard-to-reach areas.

In rural Ethiopia, only 21.2% of women delivered by skilled attendants as the 2016 EDHS report. Similarly, in the South Nation Nationalities Peoples Region of Ethiopia, more than three fourth (78.6 %) of women gave birth without a skilled birth attendant [19].

In Southern Ethiopia, within three only 10% of deliveries occurred in health institutions with MMR of 489 per 100,000 live births from which 83% of death occurred at home which is affected by poor road access and illiteracy of the husbands [20].

In the developing world, the probability of death among MWH users is 80% less than in non-users and 73% less occurrence of stillbirth among users [21]. In Eritrea, deliveries among maternal waiting home users are increased by 56% with no maternal death reports, making it a successful strategy to reduce maternal and perinatal mortality [22]. Similarly in Ethiopia, MMR was lower in users of MWHs; 89.9 per 100 000 live births compared to nonusers (1333.1 per 100 000 live births) and also users of MWHs had low stillbirth rates remained 17.6 per 1000 births compared to 191.2 per 1000 births in nonusers [23], and also 98.8% of Maternity Waiting Area users delivered with a low proportion of bad obstetric outcomes than nonusers [24].

An absence of MWH could also contribute to the low utilization of skilled birth attendants (SBA) thus women who visit health facilities in the early stages of labour were often go back home due to lack of maternity waiting areas. Hence such conditions can also adversely affect the insight of women towards institutional delivery [12].

Although the evidence showed MWHs as a strategy to reduce MMR and stillbirth, there is limited data on the level of utilization in Ethiopia and the intension of pregnant women to use this service. A little study has been conducted in Ethiopia on the intention of women to use maternity waiting homes. So, this study was directed to assess the intention and associated factors of the utilization of maternity waiting homes.

**METHODS**

**Study Area and Period**

This study was conducted in the Kamba district from March 10- April 10 /2019. Kamba district is one of the districts’ in SNNPR of Ethiopia, part of the Gamo Gofa Zone. It is located 615 km far from Addis Ababa, 390, and 110 km from the capital of SNNPR, Hawassa, and Arba Minch, capital of Gamo Gofa Zone respectively. According to the 2007 national census conducted by the Central Statistical Agency of Ethiopia, this district has a total population of 155979, of whom 79273 and 76706 are men and women respectively; 4072 or 3.02% are urban inhabitants. And a total of 30,180 households were counted in this district, resulting in an average of 4.4 persons to a household, and 29,565 housing units. Regarding health facility coverage, Kamba district has one district hospital, 7 health centers each with MWHs, 2 Satellite clinics, and 39 Health Posts. The estimated number of pregnant women in the area was 7163. There are 38 rural and 5 urban administrative kebeles found in this district. Of these, the study was conducted on randomly selected 11 kebeles [Fig.2].

**Study Design and population**

A community-based cross-sectional study was conducted using all pregnant women who were living in Kamba district as a source population, and all registered pregnant women in the selected kebeles of Kamba district as study populations, who lived at least six months in the selected kebeles before the time of the actual data collection.

**Sampling and study variables**

The assumptions used to calculate the maximum sample size for this study were population proportion of 57.3% which was taken from a study done in Jimma district [26] with a 95% confidence level (CI) and an estimated margin of error 5%. Then sample size was determined using a single population proportion formula as follows.

𝐧 = 𝐳𝛂2 (𝟏 − 𝐩)/𝐝²

𝐧 = (𝟏.𝟗𝟔)2 (𝟎.𝟓𝟕𝟑 )(𝟎.𝟒𝟐𝟕)/(𝟎.𝟎𝟓)²

n= 𝟑𝟕𝟔

With none-response rate of 10% which yields 38, the minimum required sample size = 38 + 376= 414. Since the sampling technique was multistage, the design effect of 1.5 was considered, and the final total sample size was 621. To run the Multistage sampling technique, first, from 43 Kebles in the district, 11 were selected by using simple random sampling (lottery method) technique then the proportional allocation was made for each selected kebeles. After obtaining the registration number of pregnant women from health extension workers (HEWs) of each selected kebeles, a simple random sampling technique was applied by using the registration book of pregnant women as a framework to select 621 pregnant women who fulfilled the inclusion criteria [Fig. 2].

**Socio-demographic characteristics** (age, marital status, religion, educational Status, and Occupation), **Obstetric history** (Current ANC visit, Number of ANC visit, Previous childbirth history, place of delivery, and past experience of MWH)**,** and **Psycho-social/behavioral variables** (Direct and indirect attitude, Direct and indirect subjective norm, and also direct and indirect perceived behavioral control) were included in the data collection tool [Fig. 1].

**Operational Definitions**

**Intention to use Maternity Waiting Home (MWH):** an indication of pregnant women willingness, and how much effort they are planning, and exert to utilize it. It was measured by question containing five points Likert scale. Lastly, it was dichotomized into two groups using the mean score. Those who scored above the mean (12.61) were classified as intended to use MWH, whereas those who scored less than or equal to the mean score (12.61) were considered as not intended to use MWH [25].

**Attitude towards MWH:** The degree of pregnant women’s maternity waiting home utilization behavior is influenced by her emotions, motivations, perceptions, and thoughts. It was measured by using four questions containing a five-point Likert scale. And they classified into two by using mean as they have favorable and unfavorable attitudes.

**Favorable attitude:** The respondent’s attitude score > mean (11.59), and

**Unfavorable attitude:** The respondents’ attitudes score less than or equal to the mean score **[**25]

**Indirect attitude:** It was computed by multiplying behavioral beliefs of pregnant women concerning, each outcome by corresponding outcome evaluation ratings, and then summing these product scores across all outcomes. Finally, the mean was used to dichotomize into favorable and unfavorable indirect attitude [33].

**Subjective norm:** Pregnant women’s perception of using maternity waiting homes. It was measured by four questions containing a five-point Likert scale, and they classified into two by using mean as they have favorable and unfavorable subjective norms. Those who scored above the mean (10.70) were classified as having favorable subjective norms, but those who scored less than or equal to the mean were classified as having unfavorable subjective norms [25].

**Indirect subjective norm**: It was computed by multiplying pregnant women’s normative belief about each referent by her motivation to obey with that referent and then summing these product scores across all referents. Finally, the mean score was used to dichotomize [33].

**Perceived behavioral control:** Each pregnant women’s belief concerning how easy or difficult to use maternity waiting homes. It was measured by four questions containing a five-point Likert scale and they classified into two by using mean as they have favorable and unfavorable perceived behavioral control. Those who scored above the mean (11.75) were classified as having favorable perceived behavioral control, and those who scored less than or equal to 11.75 were classified as having unfavorable perceived behavioral control [25].

**Indirect perceived behavioral control:** It was computed by multiplying each control belief of pregnant woman by her corresponding perceived power (impact) scores and then summing these product scores across all control factors, and finally those who scored above the mean was taken as having favorable indirect perceived behavioral control [33].

**Data Collection Tools and Procedures**

Data were collected using structured and pre-tested questionnaires through face-to-face interviews. Five trained data collectors (HEWs) who were recruited from health posts and three supervisors (BSc. Nurses) from health centers were executed the data collection.

**Data Quality Control**

To assure data quality, a pre-test was conducted on 5% of the total sample size among pregnant women in one of the non-selected Kebele before the actual data collection. The training was given for data collectors and supervisors on the objective, purpose of the study, respondent’s right, the confidentiality of information, informed consent, and techniques of the interview. The questionnaires were translated from English to Amharic and then to Gamogna (local language) and back to English by four language experts to assure consistency. Finally, data were entered into Epi data version 3.1, and before conducting any analysis; the data preprocessing tasks like data cleaning, coding and recording, variable re-categorization, and identification for inconsistencies were done.

**Data Analysis and Process**

First, data were coded and entered into Epi Data version 3.1 and then exported to SPSS version 24 statistical package for further analysis. Data cleaning was performed to check for frequencies, and missed values then descriptive analysis such as proportions, percentages, means, and measures of dispersion, tables, and graphs were used to describe the data. To test the association between independent and the outcome variable, logistic regression analysis was done. All variables at p-value less than 0.25 in bivariate analysis were entered to multivariate analysis to identify the independent association of variables with the intention to use MWH. Finally, significant independent associations were declared at a P-value of less than 0.05 with 95%CI.

Hosmer-Lemeshow model fitness test was used to indicate the goodness of the final model.

**Ethical Considerations**

Before data collection, Ethical approval was primarily obtained from Debre Markos University research ethical approval committee. Likewise, an official ethical clearance letter was obtained from the Kamba district health bureau. Following an explanation of the purpose of the study, consent was obtained from each participant. Also, confirmation was made that they are free to withdraw and discontinue participation without any form of prejudgments. Confidentiality of information and privacy of participants was assured for all the information they provided.

**RESULTS**

**Socio-Demographic Characteristics**

A total of 605 pregnant women participated in the study, with an overall response rate of 97.4%. The mean age of pregnant women who participated in this study was 25.95 with SD ± 4.992. About 33.4% of respondents were found in the age range of 20-24 years. The majority, 562 (92.89%) of respondents were married and 389 (64.3) of them were housewives. Furthermore, about thirty percent of respondents were completed primary education [Table1].

**Maternal Health Service Utilization of Pregnant Women**

More than half of respondents had greater than three pregnancy experiences and majorities (84.79%) of them had given birth before the current pregnancy. Likewise, about 61.60 % of the respondents gave their previous childbirth at health institutions [Table 2].

**Past Experience in Maternity waiting homes utilization**

Less than a quarter of the respondents, 130(21.49%) had past experiences on MWH utilization. Of those who utilized previously, 72(55.38%) have stayed in the service center for two weeks and only 9(6.92%) of them have stayed for more than two weeks [Table 3].

**Intentions to Use Maternity Waiting Home**

Less than half, 295 (48.76%) with 95% CI (47%-55%) of respondents were intended to use maternity waiting homes [Fig. 3].

**Descriptive statistics of components of Theory of Planned Behavior (TPB)**

**Direct components TPB**

Two hundred seventy-three (45.12%) of the pregnant women had a favorable attitude to use MWH with a mean of 11.59 (SD ± 4.349). And also, Two hundred forty-nine (41.16%) and 327 (54.05%) of the respondents had favorable subjective norm and perceived behavioral control with a mean of 10.70 (SD ±4.565) and 11.75 (SD ±3.723) respectively [Fig.4].

**Indirect TPB components**

**Indirect attitude**

Three hundred eighty-six (63.8%) of pregnant women had favorable indirect attitudes with a mean score of 71.75%. Four hundred seventy-six (78.68%) of pregnant women agreed that MWH would help them to get a healthy child. Likewise, 500 (82.64%) of them agreed MWH would help them to be happy & reduce labour fear. Additionally, 352 (58.18%) and three hundred eleven (51.40%) of the respondents mentioned that staying at MWH is very good to get a healthy child and to reduce labour fear respectively.

**Indirect subjective Norm**

Two hundred thirty-six (39%) of the respondents had favorable indirect subjective norms with a mean score of 31.28%. Three hundred seven (50.75%) of them agreed that HEWs thinks that they should stay in MWH. Two hundred twelve (35.05%) them agreed that their husbands think that they should stay in the maternity waiting home. In The motivations to comply with the above normative beliefs, 299 (49.42%) of respondents reported that HEWs approval for their stay in MWH was much important. Of them, less than a quarter 128 (21.15%), and 111 (18.34%) were reported that their mothers' and husbands' approval to use MWH is much important respectively.

**Indirect perceived behavioral control**

Three hundred nine (51.1%) the respondents had favorable indirect perceived behavioral control with a mean score of 56.6%. The majority 495(81.82%) of them reported that it's unlikely to get enough food in MWH to use it, followed by 437 (72.24%) of them reported they were unlikely to get transportation/walk to long distance. And also 379 (62.60%) pregnant women reported it’s unlikely to get individuals to take care of their family while they staying in MWH. The power of control belief result revealed that 475 (78.52%) and 447 (73.88%) of them agreed that the availability of food and transport accessibility makes it easy to use MWH respectively.

**Bivariate and Multivariate Analysis of Factors Associated with Intention to use Maternity Waiting Home**

In multivariate regression analysis, Occupation(government employees), previous child birth history, past experience of MWH, direct and indirect subjective norm and perceived behavioral control of the respondents were the factors that significantly associate with Intention to use Maternity Waiting Home [table4].

**DISCUSSION**

This study was conducted to identify the Intention to use maternity waiting home and associated factors among pregnant women in Kamba district, Gamo Gofa zone, southern Ethiopia.

According to this study, 48.76% (95%CI: 47-55%) of pregnant women were intended to use maternity waiting homes, which is consistent with a previous study conducted in Mettu district, Illubabor zone, Ethiopia which is 48.80 % [ 25].

However, this finding is lower than other previous studies conducted in Jimma district, Ethiopia, which was 57.3% [26] and Butajira, southern Ethiopia, it was 55.1% (27) and a study conducted in Kenya which was 61.1% [30]. These discrepancies could be due to variations in socio-demographic differences among the study areas; disparities in the health service utilization and accessibility; a gap of knowledge about MWH services among the study populations and also the nature of the study. This 48.76% of intention to use maternity waiting home is greater than prior studies conducted in rural Health Centers of Ethiop which was 27% [31], and a study conducted in rural Kenya which revealed 45% of women intended to use maternity waiting homes [28]. The reasons for this discrepancy might be due to variations in the study period, and the study population. In this study, the occupation of pregnant women was found to be one of the significantly associated variables with the intention to use MWH. Consequently, respondents who were government employers were 2.87 times (AOR: 2.87, 95%CI: 1.54-5.36) more likely intended to use MWH as compared to housewives. Nonetheless, there has been limited scientific evidence concerning this significant association, and the possible reason for this association could be due to those women who are government employees might have exposure to information and better insight about MWH services as compared to those women who are housewives.

Pregnant women who had previous childbirth history were 2.1 times (AOR: 2.08, 95%CI: 1.22-3.57) more likely intended to use MWH than those pregnant women who had not given birth before. Regarding this significant association between previous childbirth history and intention to use maternity waiting homes, evidence has been limited, though the possible reason for this association could be those women who had a history of previous childbirth might have better information about the availability and importance of MWH services during previous health facility visiting than those not ever gave birth.

According to the findings of this study, pregnant women who had past experience in MWH utilization were 4.35 times (AOR: 4.35, 95%CI: 2.63-7.18) more likely intended to use MWH as compared to those who have not utilized it in the past. This finding is supported by a study done in Jimma district which revealed that women who had past experience of MWH were 16.3% more intended to use MWH than those who do not have past experience (26). The possible reason for this significant association could be; those women who utilized MWH in the past might have better insight into the benefit of MWH service as compared with women who were not ever utilized.

This study revealed that direct subjective norm was significantly associated with intention to use maternity waiting home. As a result, pregnant women with favorable direct subjective norm were 57% (AOR: 1.57, 95%CI: 1.01-2.47) more likely intended to use MWH as compared to those with the unfavorable subjective norm which is congruent with a study done in Mettu districts, Ethiopia which depicted that women who had favorable subjective norm were 2.8 times more likely intended to use MWH than their counterparts, and also it is compatible with a study conducted in Jimma district (25, 26). This could be due to the crucial importance of significant others of pregnant women in the decision-making process regardless of her own willingness to use MWH among those having a favorable attitude.

This study also revealed that indirect subjective norm has a significant association with intention to use MWM i.e. respondents who have favorable indirect subjective norm were 2.2 times (AOR: 2.18, 95%CI: 1.38-3.44) more likely intended to use MWH as compared to those who have unfavorable indirect subjective norm and it is supported with a study done in Jimma district, Ethiopia (26). In the current study, 50.75% and 35.05%) of the respondents agreed that HEWs and their husbands think that they should stay in MWH.

This finding is congruent with a study conducted in Jimma district, Mettu District, and rural Zambia in which HEWs and husbands have participated in the decision to use MWHs (16, 25, and 26). The reason for this association could be due to the valuable significance of HEWs and husbands' role towards the utilization of MWH of those pregnant women.

The finding from this study showed that both direct and indirect perceived behavioral control have a significant association with intention to use MWH i.e. pregnant women who had favorable direct were 3 times (AOR: 3.00, 95%CI: 2.03-4.43) more likely intended to use MWH as compared to those with unfavorable direct and indirect Perceived behavioral controls respectively. This is compatible with a finding from Mettu district in which participants with favorable perceived behavioral control were 99% more likely intended to use MWH as compared to unfavorable perceived behavioral control (25), and also it is congruent with a survey was done in Jimma, Ethiopia which shown that direct perceived behavioral control had a significant association with intention to use MWH (26). Similarly in this survey, indirect attitude showed significant association with intention to use MWH in addition to direct attitude.

Pregnant women with favorable indirect perceived control were 84% more (AOR: 1.84, 95%CI: 1.25-2.70) likely intended to use MWH as compared to their counterparts. This finding is also congruent with a survey done in Jimma which showed a strong significant association between indirect perceived behavioral control and intention to use MWH (26). The potential rationale for this evenness could be due to parallel perceived barriers such as long-distance/lack of transportation, unavailability of food in MWH among the two populations since both of them were conducted among rural districts. This study also revealed 81.82% of the respondents reported that it's difficult to get enough food in MWH during service utilization it followed by 72.24% of them reported they were unlikely to get transportation or walk to long distance. This finding is greater than the study conducted in Butajira in which 41.8% and 33.4% of participants were unlikely to get enough food and transport to go to MWHs respectively (27). The differences could be due to time variation, and socio-demographic differences between the two populations.

The community-based nature of this study is its strength, however, as this is a quantitative study, it might lack some hidden attitudes of women that can predict the intention and which would be addressed by qualitative type of studies and it is tough to declare cause-effect relationship between the outcome and dependant variables due to the cross-sectional nature of this study.

**CONCLUSION**

Generally, the intention to use maternity waiting home among pregnant women is less than half, indicated that the magnitude of intention is not satisfactory. This study also identified the occupation of respondents, history of previous childbirth, past experiences of MWH utilization, direct and indirect subjective norm, and perceived behavioral control as significantly associated factors affecting intention to use MWH.

**RECOMMENDATIONS**

Since the magnitude of intention to use MWH among pregnant women in this study is unsatisfactory, it calls for the intervention of the community as depicted below.

1. It is better to mobilize the community and provide health education, concerning MWH service to all the community and mothers to improve the subjective norm.
2. Community based stakeholders such as health development armies need to be strengthened and extended to alleviate transportation problems and food shortages, those upshot women with unfavorable perceived control beliefs, through integration with local and Federal governments to upsurge perception of mothers towards behavioral controls.
3. Health care professionals need to advice and counsel the mothers on MWH service at ANC visiting to increase their intention
4. In this area, it is better to do further researches in a qualitative approach

**Abbreviations /Acronyms**

**AOR:** Adjusted Odds Ratio, **ANC:** Antenatal Care, **EDHS:** Ethiopian Demographic Health Survey, **EmOC:** Emergency Obstetric Care, **HEW:** Health Extension Worker, **MM:** Maternal Mortality, **MMR:** Maternal Mortality Ratio, **MWH:** Maternal Waiting Home, **NGO:** Non-Governmental Organization, **SBA:** Skilled Birth Attendant, **TPB:** Theory of Planned Behavior, and, **TRA:** Theory of Reasoned Action

**Declarations**

**Acknowledgments:** The authors would like to thank data collectors and supervisors for their assiduousness and dedication in collecting and inputting high-quality data and also we would like to thank the study participants for their willingness to participate, kindly provision of the necessary information, and scarification of their valuable time.

**Funding**

This research received no specific grant from any funding agency.

**Availability of data and materials**

Supporting data for the current study are available from the corresponding author on a reasonable query.

**Ethical Approval and Consent to the participant**

Ethical approval was primarily obtained from Debre Markos University college o health sciences research ethical approval committee. Likewise, an official ethical clearance letter was obtained from the Kamba district health bureau. Following an explanation of the purpose of the study, informed consent was obtained from each participant. Anonymity and confidentiality were ensured for information.

**Consent for publication**

Not applicable.

**Competing interests**

The authors declare that they have no competing interests.

**Authors’ Contributions**

All authors contributed to data analysis, drafting or revising the article, gave final approval of the version to be published, and agree to be accountable for all aspects of the work

**REFERENCES**

1. World Health Organization. Division of reproductive health Safe Motherhood, 1996
2. Julie M. Buser KP, Michelle L. Munro-Kramer, Jody R. Lori. Cost Analysis of Maternity Waiting Homes in Rural Liberia. Africare 2018
3. Khan N, Naziri M, Rafique N, Mahmud R, Rafique N, Ahmadzai M, et al. UNICEF: Innovative Approaches to Maternal and Newborn Health. Maternal, Newborn and Child Health, 2013
4. Fogliati P, Straneo M, Mangi S, Azzimonti G, Kisika F, Putoto G. A new use for an old tool: maternity waiting homes to improve equity in rural childbirth care. Results from a cross-sectional hospital and community survey in Tanzania. Health policy and planning, 2017; 32(10):1354-60.
5. Odusola A. Accelerating Progress On Maternal Health In Africa: Lessons From Emerging Policy And Institutional Innovations Global MDG Conference, UNDP Working Paper No 11, UNDP Publishing. 2013
6. Kwamena Sekyi Dickson KSA, Amu H. What Influences Where They Give Birth? Determinants of Place of Delivery among Women in Rural Ghana, International Journal of Reproductive Medicine, 2016:1-8
7. Cephas Sialubanje KM, Elisa Maria Kirch, Marit S.G. van der Pijl Davidson H. Hamer Robert A.C. Ruiter, Husbands’ experiences and perceptions regarding utilization of maternity waiting homes in rural Zambia, 2014.
8. Jody R. Lori, Anna C. Wadsworth, Michelle L. Munro, and Sarah Rominski, Promoting access: The use of maternity waiting homes to achieve safe motherhood. Midwifery, 2013; 29 1095–102.
9. Elisa M. Kirch MSP. Maternity waiting homes in Kalomo, Zambia. Share Nate. 2014.
10. Yemisrach B. Okwaraji EL. Barriers in physical access to maternal health services in rural Ethiopia. BMC health services research, 2015; 15:43.
11. Asheber Gaym LP, Khynn Win Win Soe. Maternal Waiting Home in Ethiopia, Three Decades Experiences, Ethiopian Medical Journal, 2012; 50(3):209-19.
12. Bedford J GM, Admassu M,Girma A. 2012 A normal delivery takes place at home’: A qualitative study of the location of childbirth in Rural Ethiopia. Maternal Child Health, 2012
13. Ethiopian Emergency Obstetric and Newborn Care (EmONC) Assessment. Averting Maternal Death and Disability (AMDD), Columbia University New York, USA, 2016:278-82.
14. Eckermann Elizabeth GD. Maternity waiting homes in Southern Lao PDR: the unique 'silk home. Journal of obstetrics and gynecology research, 2008; 34(5):767-75.
15. Nancy A. Scott TV, Jeanette L. Kaiser, Thandiwe Ngoma, Kaluba Mataka, Elizabeth G. Henry, Godfrey Biemba, Mary Nambao, Davidson H. Hamer. Listening to the community: Using formative research to strengthen maternity waiting homes in Zambia, PLoS ONE, 2018; 13(3):1 16.
16. Cephas Sialubanje KM, Davidson H. Hamer, Robert A. C. Ruiter. Personal and environmental factors associated with the utilization of maternity waiting homes in rural Zambia. BMC Pregnancy and Childbirth, 2017; 17:316.
17. World Health Organization (WHO). Trends in maternal mortality: 1990 to 2015: estimates by WHO, UNICEF, UNFPA, World Bank Group and the United Nations Population Division Geneva, 2015.
18. Transforming Our World: The 2030 Agenda for Sustainable Development. 2015.
19. Ethiopian Demographic and Health Survey Federal Democratic Republic of Ethiopia, Addis Ababa. 2016.
20. Yaya TD, Lindtjørn B. Maternal Mortality in Rural South Ethiopia: Outcomes of Community-Based Birth Registration by Health Extension Workers PLoS ONE 2015; 10(3):1-17.
21. Dadi TL, Bekele BB, Kasaye HK, Nigussie T. Role of maternity waiting homes in the reduction of maternal death and stillbirth in developing countries and its contribution for maternal death reduction in Ethiopia: a systematic review and meta-analysis. BMC health services research. 2018; 18(1):748.
22. Andemichael BH, Kosia K. Maternity waiting homes: A panacea for maternal and neonatal conundrums in Eritrea. Journal of the Eritrean Medical Association, 2009; 14(1):18-29.
23. Kelly J KE, Poovan P, Schiffer R, Redito A, Winter H, MacArthurb C. The role of a maternity waiting area (MWA) in reducing maternal mortality and stillbirths in high-risk women in rural Ethiopia, BJOG: an international journal of obstetrics and gynecology. 2010; 117(11):1377–83.
24. Meshesha B DG, Hailemariam T: The Role of Maternity Waiting Area in Improving Obstetric Outcomes: A Comparative Cross-sectional Study, Jinka Zonal Hospital, in Southern Regional State. Journal of Women’s Health Care, 2017; 6(6):1-8.
25. Yismaw W. Intention to Use Maternity Waiting Home among Pregnanat Women in Mettu District, Southwestern Ethiopia, 2018
26. Bayih G, Tamirat A. Intention to use Maternity Waiting Home among Pregnant Women in Jimma District, Southwest Ethiopia. Global Journal of Medical Research, 2016; 16 (6)
27. Vermeiden T, Medhin G, Gaym A, van den Akker Th, Stekelenburg J. Factors associated with intended use of a maternity waiting home in Southern Ethiopia:. BMC Pregnancy and Childbirth 2018; 18(38):1-9.
28. Mramba L, Ondieki C, Kimang D. Reasons for low utilization of a maternity waiting home in rural Kenya. International Journal of Gynecology and Obstetrics, 2009; xxx: xxx–xxx.
29. Rukia Ware Abdulkadir. Awareness, Attitude towards, and Utilization of Maternity Waiting Home by Mothers in Merti Sub County, Isiolo County, 2017
30. Oyoko PO. Factors Influencing Utilization of Maternity Waiting Home in Kenya: A case of Turkana Westsub-County, 2018
31. Tadele G, Tiruneh BWT, Karim AM, Aklilu W, Zemichael NF, Gebrekirstos T, Tekle E. Maternity waiting homes in Rural Health Centers of Ethiop: The situation, women’s experiences and challenges. Ethiop J Health Dev. 2016; 30(1):19-28.
32. Zhang K. Theory of Planned Behavior: Origins, Development &Future Direction. International Journal of Humanities &Social Science Invention (IJHSSI), 2018; 7 (05):76-83.
33. Daniel E. Montaño DK. Theory of Reasoned Action, Theory of Planned Behavior, and the Integrated Behavioral Model: Karen Glanz Barbara K. Rimer K. Viswanath: Health Behavior and Education Theory, Research, and Practice C. Tracy Orleans	2008	pp. 67-92.
34. Theory of Planned Behavior: <http://people.umass.edu/aizen/tpb.html>.
35. Icek A. From intentions to action: a theory of planned behavior. In J Huhl & J Beckman (Eds), Will; performance; control (psychology); motivation (psychology), 1985
