# Supplementary material for: Intention to use maternity waiting home and associated factors among pregnant women in Gamo Gofa zone, Southern Ethiopia, 2019
Source: PLoS One. 2021 May 13;16(5):e0251196. doi: 10.1371/journal.pone.0251196 (PMC8118329; doi:10.1371/journal.pone.0251196)
Supplement: Attachment — Submitted filename: Response to Reviewers.docx [file pone.0251196.s011.docx]

**PONE-D-20-31704**

**Intention to use maternity waiting home and associated factors among pregnant women in Gamo Gofa zone, Southern Ethiopia, 2019**

**Dear Frank T. Spradley**

**Academic Editor of PLOS ONE journal**

We Authors would like to thank you for your constructive comments, and suggestions. In accordance with the reviewers’ comments, we made a revision in the whole document based on PLOS ONE’s publication criteria and we respond to comments and questions line by line. Therefore, we submitted a revised version of the manuscript that addresses the points raised during the review process.

**Authors’ response to the Academic Editor comments’**

When submitting your revision, we need you to address these additional requirements.

1. Please ensure that your manuscript meets PLOS ONE's style requirements, including those for file naming.

**Authors’ response:** The whole document revised based on PLOS ONE’s publication criteria

2. In your Methods section, please provide additional information about the participant recruitment method and the demographic details of your participants. Please ensure you have provided sufficient details to replicate the analyses such as:

-	a description of any inclusion/exclusion criteria that were applied to participant recruitment,

**Authors’ response:** Those pregnant women who lived less than six months in the study area, and those who delivered by caesarean section were excluded from the study.

-	a table of relevant demographic details,

**Authors’ response: The relevant demographic details are included under the study area of this study in a text form**.

-	a statement as to whether your sample can be considered representative of a larger population,

-	description of how participants were recruited, and descriptions of where participants were recruited and where the research took place.

**Authors’ response:** This study was conducted in Gamo Gofa zone in southern Ethiopia using the data of pregnant woman in Kemba district, and to run the multistage sampling technique districts in this zone were considered as clusters because there is a homogeneous feature between districts. Then, due to the number of pregnant women estimation in each district and our sample size, Kebma district was selected randomly. In Kebma district there are 43 kebeles, and to make the sample being representative to all Kebeles, we took 25% of them (11 kebeles) randomly. Additionally, we employed design effect of 1.5 to represent the sample to Gamo Gofa zone. By considering the heterogeneous feature of Kebeles within a cluster, proportional allocation of pregnant women was done in each selected stratum (Kebele) ,and in each Kebele there are assigned HEWs ,and these HEWs provide community visits at house hold level at least 2 times a week and one of their responsibility is to investigate whether pregnant woman/s is present in that hose hold or not , and then to register them. The registration includes the gestational age, EDD, Name, Phone number (if available), the exact location and others. After obtaining the registration number of pregnant women from health extension workers (HEWs) of each selected Kebele, a simple random sampling technique (lottery method) was applied by using the registration book of pregnant women as a framework. Furthermore, 621 pregnant women who fulfilled the inclusion criteria were recruited based on their registration number via simple random sampling technique, and interview was done at the household level for those randomly selected pregnant women.

3. Please include additional information regarding the survey or questionnaire used in the study and ensure that you have provided sufficient details that others could replicate the analyses. For instance, if you developed a questionnaire as part of this study and it is not under a copyright more restrictive than CC-BY, please include a copy, in both the original language and English, as Supporting Information. If the original language is written in non-Latin characters, for example Amharic, Chinese, or Korean, please use a file format that ensures these characters are visible.

**Authors’ response: The detail of the questionnaire used in this study is included in the supporting information via three languages.**

4. Please state whether you validated the questionnaire prior to testing on study participants. Please provide details regarding the validation group within the methods section.

**Authors’ response:** Face validity was conducted to assess the form of the questionnaire in terms of feasibility, readability, evenness of techniques and formatting, and the clearness of the language, that is to assess the presentation and relevance of the measuring tool as to whether the items in the tool emerge to be pertinent, logical, explicit and obvious and to validate the questionnaire, 2 experts groups on each English, Amharic, and Gamogna language were participated.

5. Please provide additional details regarding participant consent. In the ethics statement in the Methods and online submission information, please ensure that you have specified what type you obtained (for instance, written or verbal, and if verbal, how it was documented and witnessed). If your study included minors, state whether you obtained consent from parents or guardians. If the need for consent was waived by the ethics committee, please include this information.

**Authors’ response:** We obtained verbal consent from all participants, and it was documented using the following consent form.

**Consent form**

Hello my name is _________________ (name of data collector). I am____________________ (the data collector briefly introduce him/herself), and I am here to collect data on “Intention to use maternity waiting home and associated factors among pregnant women” for research purpose. The objective of this study is to assess intention to use maternity waiting home and associated factors among pregnant women in Gamo Gofa zone. The benefit of your participation in this study is to improve maternal and neonatal health in your community as well as a country as a whole by increasing institutional delivery. Hence, your trustworthy and frank participation is ultimately important to achieve this goal. All the information that you provide must be kept confidentially, and your name and information will not be disclosed. The information you give is only disclosed to the investigators and they will use it only for this research purposes. You’ve a full right to not respond to all or part of the questions.

Are you voluntary to participate in this study? 1. Yes	2. No	Thank you!!

**The name of Data collector: ____________________**

**Cell phone of data collector:**

**Date of data collection**:

6. Your ethics statement should only appear in the Methods section of your manuscript. If your ethics statement is written in any section besides the Methods, please delete it from any other section.

**Authors’ response:** The ethics statement which was appear in declaration section is deleted

7. Please include your tables as part of your main manuscript and remove the individual files. Please note that supplementary tables should be uploaded as separate "supporting information" files.

**Authors’ response: Revision is made based on your comments**

**Response to Reviewers’**

Reviewer #1: General comments

**1. General comments**

1.1: Authors should read thoroughly the author’s submission guidelines and strictly follow it for all sections of the manuscript. For this purpose they should download the sample pdf of abstract and sample pdf of manuscript. They should strictly follow the guideline for font size, writing style – upper and lower case writing in title, affiliation, abstract, main manuscript, table titles, figure titles, and references.

**Authors’ response:** We revised the whole document based on the PLOS ONE’S publication criteria

1.2: While submitting manuscript authors did not make the manuscript in double space, page numbers and line numbers.

**Authors’ response:** We made a revision, and we include page numbers and line numbers.

2. English language has to be improved.

**Authors’ response:** We tried to correct all grammatical, spelling, punctuation, and preposition errors in the whole manuscript with language expert.

**2. Title**

2.1: Page (P) 2, lines (L) – 7, 9, While writing affiliation, instead of writing College of Health sciences, it should be College of Health Sciences.

**Authors’ response:** we corrected it as **College of Health Sciences**

2.2: P2, L10 – it should be Department of Public Health, College of Health Sciences

**Authors’ response:** we corrected it as **Department of Public Health, College of Health Sciences**

**3. Abstract**

3.1: P4, L56 – research aim is not the section in abstract. Merge it in background.

**Authors’ response:** Research aim is merged in the background

3.2: P4, L59 – delete add comma after April 10, and delete /

**Authors’ response:** It is corrected as **from March 10 to April 10, 2019**

3.3: P4, L60 – it should be questionnaire and interview.

**Authors’ response:** It is corrected as a **questionnaire** and **interview**.

3.4: P4, L66 – it should be 95%.

**Authors’ response:** It is corrected as **95%**

3.5: P4, L68 – it should be (AOR

**Authors’ response:** **AOR** is included

3.6: P 4, L72 – Recommendation is not there – follow guidelines

**Authors’ response:** Recommendation is deleted

3.7: P4, L77 – Delete Kemba district and write Ethiopia. For writing Keywords – they should match with MeSH terms.

**Authors’ response:** Kemba district is deleted, and the word Ethiopia	is included

**4. Background**

4.1: P5, L79 – It should be World Health Organization

**Authors’ response**: It is corrected as **World Health Organization**

4.2: P5, L81 – It should be health facility instead of hospitals. In your study area there are primary health care units. They are not hospitals.

**Authors’ response:** It is corrected as **health facilities**

4.3: P5, L93 – It should be long distance instead of several distances.

**Authors’ response:** It is corrected as **long distance**

4.4: P5, L98 – it should be population instead of populations.

**Authors’ response:** It is corrected as **population**

4.5: P5, L101 – give long form of SNNPR as it is appearing first time. It should be Oromia instead of Oromo region. Add and after (56%); and least

**Authors’ response:** It is corrected as **Southern Nations, Nationalities, and Peoples' Region (SNNPR) (57%) and Oromia region (56%); and least (8%) in the Gambella region**

4.6: P5, L133 – Add per – it should be as --- attendants as per the 2016.

**Authors’ response**: the preposition **per** is added

Comment: Authors did not cite the following publications to add in the background or discussion

a. Kurji J, Gebretsadik LA, Wordofa MA, Sudhakar M, Asefa Y, Kiros G, Mamo A, Bergen N, Asfaw S, Bedru KH, Bulcha G, Labonte R, Taljaard M, Kulkarni M. Factors associated with maternity waiting home use among women in Jimma Zone, Ethiopia: a multilevel cross-sectional analysis. BMJ Open. 2019 Aug 28;9(8):e028210. doi: 10.1136/bmjopen-2018-028210. PMID: 31467047; PMCID: PMC6720516.

b. van Lonkhuijzen L, Stekelenburg J, van Roosmalen J. Maternity waiting facilities for improving maternal and neonatal outcome in low-resource countries. Cochrane Database Syst Rev. 2012 Oct 17;10(10):CD006759. doi:10.1002/14651858.CD006759.pub3. PMID: 23076927; PMCID: PMC4098659.

c. Swanson DL, Franklin HL, Swanson JO, Goldenberg RL, McClure EM, Mirza W, Muyodi D, Figueroa L, Goldsmith N, Kanaiza N, Naqvi F, Pineda IS, López-Gomez W, Hamsumonde D, Bolamba VL, Newman JE, Fogleman EV, Saleem S, Esamai F, Bucher S, Liechty EA, Garces AL, Krebs NF, Hambidge KM, Chomba E, Bauserman M, Mwenechanya M, Carlo WA, Tshefu A, Lokangaka A, Bose CL, Nathan RO. Including ultrasound scans in antenatal care in low-resource settings: Considering the complementarity of obstetric ultrasound screening and maternity waiting homes in strengthening referral systems in low-resource, rural settings. Semin Perinatol.

2019 Aug;43(5):273-281. doi: 10.1053/j.semperi.2019.03.017. Epub 2019 Mar 16. PMID: 30979599; PMCID: PMC6597951.

d. Buser JM, Lori JR. Newborn Outcomes and Maternity Waiting Homes in Low and Middle-Income Countries: A Scoping Review. Matern Child Health J. 2017 Apr;21(4):760-769. doi: 10.1007/s10995-016-2162-2. PMID: 27475822.

e. Gaym A, Pearson L, Soe K. Maternity waiting homes in Ethiopia – three decades experience…..

**Authors’ response:** The listed citations included in the discussion part of the manuscript **and this reference (**Gaym A, Pearson L, Soe KW. Maternity waiting homes in Ethiopia--three decades experience. Ethiopian medical journal, 2012 Jul;50(3):209-19) is reference number 13 currently and it was reference number 11 previously. But I can’t cite the result of the research, which is entitled by Newborn Outcomes and Maternity Waiting Homes in Low and Middle-Income Countries focusing on the impact of MWHs on neonatal health.

**5. Methods**

Comment:

a. Follow guideline for section and sub-sections.

**Authors’ response:** As per the guideline for section and sub-sections, we made revision.

5.1: P8, L – 170, make it as April 10, and delete /

**Authors’ response:** It is corrected as **from March 10 to April 10, 2019**

5.2: P8, L – 171, Authors should decide whether it should be as Gamo Gofa Zone or it should be used as Gamo Gofa zone in the whole manuscript including titles of tables and figures. Also authors should decide whether the percentages should be presented with one digit or two digit e.g. 3.2 or 3.21. These percentages be consist in the whole manuscript including tables and figures.

**Authors’ response: we used Gamo Gofa zone in the whole document**

5.3: P8, L – 174 and 175, Instead of actual figures of population it can be written as about 160,000 and % male and female.

**Authors’ response:** The preposition **about** is included**.**

5.4: P8, L – 190 – 194 either delete this or put it in supplementary information. This formula is not required.

**Authors’ response: the formula is deleted**

5.5: P9, L – 198, Explain what is Kebele – International reader will not understand.

**Authors’ response:** We explained it as **Kebeles (the smallest administrative units of Ethiopia)**

5.6: P9, L – 203, Fig.2 - This figure can go into supplementary information.

**Authors’ response:** It is put in the supplementary information

5.6: P9 – There is needed to link these two paragraphs giving information about data collection details. (L – 203 and L – 204).

**Authors’ response**: we create the link by using **and variables such as**

5.7: P9 – Line – 208. Write as insert Fig. 1 here.

**Authors’ response:**

5.8: P10 – Line – 225. Write value of mean in the bracket. It should read – the mean () was to ----------

**Authors’ response:** mean (71.75%) is included

5.9: P10 – Line – 234. Write value of mean in the bracket. It should read – the mean () was to ----------

**Authors’ response:** mean (31.28%) is included

5.10: P10 – L 237 – Instead of scale and they – it should be scale and were

**Authors’ response:** It is corrected as **scale, and were**

5.11: P10 – L 247 – 248 – it should be --- health posts who were supervised by three supervisors (B.Sc, Nurses) from health centre.

**Authors’ response: It is corrected as** from health posts who were supervised by three supervisors (BSc, Nurses) from health centers were executed the data collection.

5.12: P11 – L 270 – Why this statement is required. Earlier you have not described this model. Or describe it little bit it here.

**Authors’ response:** We put this model because we checked the goodness of the final model using the value of Hosmer-Lemeshow model fitness test, and we decided that the final model is best fitted when the value is insignificant that is greater than 0.05.

6. Results

Comment: Follow author’s guidelines for paragraph and sub-paragraphs font size, fond type, etc. Also decide number of decimals one or two to be presented percentages in the manuscript and Tables, and figures. It should be consistent.

**Authors’ response:** Revision is made based on the guideline

6.1: P12 - L – 286 – Type – Insert table 1 here

**Authors’ response: Table 1 is inserted**

6.2: P12 - L – 290 - Type – Insert table 2 here

**Authors’ response: Table 2 is inserted**

6.3: P12 - L – 294 - Type – Insert table 3 here

**Authors’ response: Table 3 is inserted**

6.4: P12 - L – 297 - Type – Insert Figure 3 here

**Authors’ response: It is included in the supplementary information**

6.5: P12 - L – 303 - Type – Insert Figure 4 here

**Authors’ response: It is inserted and labeled as Figure 2 because figure 2 and 3 included in the supplementary information**

Comment: Your result section description is short. In bivariate and multivariate section you bring AOR descriptions here from discussion section. In discussions do not put analysis figures. Give probable reasons, compare with other studies.

**Authors’ response:** we made a description of the bivariate and multivariate analysis in the result section

**7. Discussions**

Comment: Discussion is quite long.

**Authors’ response: We tried to minimize it.**

7.1: P14 L – 338 – Instead of Intention it should be intention.

**Authors’ response: It is corrected as intention**

7.2: P15 L – 373 – 374 – It should be --- women who did not use it.

**Authors’ response: It is corrected as women who did not use it**

7.3: P16 L – 391 – 392 – It should be --- (16,25,26).

**Authors’ response: It is corrected as	(16,25,26)**

7.4: P16 L – 397 – it should be – perceived

**Authors’ response: It is corrected as	perceived**

7.5: P16 L – 414 – whether it is unlikely or likely. Check again.

**Authors’ response: We checked it and it is unlikely**

7.6: P16 L – 415 – it should be – -----socio-demographic differences and availability of road and transportation between the populations.

**Authors’ response: It is corrected as socio-demographic differences and availability of road and transportation between the populations.**

**8. Recommendation**

Comment: Follow the guidelines of journal. No separate recommendation section. It should be included in conclusions.

**Authors’ response: We corrected it based on the guidelines of the journal**

9. Declaration

9.1: P18 – L 451 – it should be sacrifice

**Authors’ response: It is corrected as sacrifice**

9.2: P18 – L 458 – Debre Markos University collage of health sciences

**Authors’ response:** It is corrected as Debre Markos University College of Health Sciences

**10. References**

Comment: Follow guidelines for references – how to write authors, and other details of article of the journal, book, chapter in book, website.

**Authors’ response: Dear reviewer based on your comments, we checked all references and we took correction for all.**

10.1: P19 – L 473 – check the authors list……

10.2: P19 – L 485 – Check authors

10.3: P19 – L 488 – check the authors

10.4: P19 – L 494 – check the author, this is not complete.

10.5: P19 – L 499 – why 2012.

**Authors’ response: we omitted it.**

10.6: P20 – L 502 – it should be – comma after Columbia University,

10.7: P20 – L 506 – 509 – No. of authors are more than 6. As Vancoure system cannot be more than 6 authors --- after 6th author it should be et.al.

**Authors’ response: We checked it online but it is as it is. Can we correct it manually?**

10.8: P20 – L 510 – What is Ruiter?

**Authors’ response: Correction is made and** Ruiter RA is one of the Authors list

10.9: P21 – L 535 – 536 – Is this master’s thesis? Which University? Title of this study and this reference is same.

**Authors’ response: It is unpublished master’s thesis from Addis Ababa University**

10.10: P21 – L 543 - what is this xxx – xxx – xxx.

**Authors’ response: revision is taken on it.**

10.11: P21 – L 545 – name of journal, vol. and page numbers

10.12: P21 – L 548 – no. of authors 6 and then et.al

10.13: P21 – L 553 – 555 – Check with reference guide. How to refer?

10.14: P21 – 556 – Is it a chapter in the book?

10.15: P21 – 557 – 558 – check how to refer chapter in book.

11. Tables

**Authors response’: we revised all tables based on the guideline**

11.1: Table 1 – titles should be as per guidelines

**Authors response’: The title is corrected as Socio-demographic characteristics of pregnant women**

11.2: Table 2 – titles should be as per guidelines. ANC visit for current pregnancy – yes no. is 386, but next variable – number of ANC visit – total number of 64, 117, 118, 89 is 388. And % are correct with 388 and not with 386.

**Authors response’: The title is corrected as maternal health services utilization among pregnant women, and ANC visit for current pregnancy is corrected as yes (388) and no (217)**

11.3: Table 3 - titles should be as per guidelines. Delete all % sign. Why one digit percentage is presented for variable – Reasons for previous utilization ---

**Authors response’: The title is corrected as Past experiences on MWH service among pregnant women and all % signs are deleted and two digit percentages are presented for a variable reasons for previous utilization of MWH.**

11.4: Table 4 – titles should be as per guidelines. Below the “*” P=< statistically associated --- should be statistically significant.

**Authors response’: The title is corrected as Bivariate and multivariate analysis of factors associated with intention to use maternity waiting home, and the other is corrected as * =p<0.05: statistically significant**

12. Figures

**Authors response’: we revised all figures based on the guideline**

12.1: Figure 1: titles should be as per guidelines.

**Authors response’: The title is corrected as Fig1. Conceptual Framework showed factors associated with intention to use maternity waiting home, and adapted from the Theory of Planned Behavior [28-35].**

12.2: Figure 2: titles should be as per guidelines. This can go as supplementary attachment.

**Authors response’: The title is corrected as S1 Fig. Diagrammatic presentation of sampling procedure used to recruit pregnant women in Gamo Gofa Zone, Southern Ethiopia, 2019, and included in the supplementary information.**

12.3: Figure 3: titles should be as per guidelines.

**Authors response’: The title is corrected as S2 Fig. Magnitude of intention to use maternity waiting home among pregnant women in Gamo Gofa zone, Southern Ethiopia, 2019**

12.4: Figure 4: titles should be as per guidelines.

**Authors response’: The title is corrected as Fig. 2 Proportion of direct components of theory of planned behavior among pregnant women in Gamo Gofa zone, Southern Ethiopia, 2019**

**Reviewer #2: It is my pleasure to be designed as a reviewer of this paper, thank you very much!**

This is a good paper that examined the intention to use MWH, one of the strategies to bridge geographic barriers to access obstetric care.

**Line by line response to reviewer#2 comments**

**Dear reviewer, we authors would like to thank you in advance for your valuable comments, and questions. And we replied for your concerns line by line.**

Comments

Background

1) The background section needs to be restructured for smooth flow from general problem to specific problem statement. In this case, please try to develop themes for each paragraph and link them logically to the issue. You may follow what is intention to use MWHs, what determines women’s intention to use MWHs (individual, community, health system factors) or what is known in the area, what is unknown, contribution of this paper to the scientific knowledge. You need also explain your theoretical framework here. Figure 1 is not described in the text

**Authors’ response: The background is restructured based on your comment, and also Figure 1 is described and cited.**

2) You use figures from 2016 EDHS data. Please update by 2019 mini-EDHS

**Authors’ response: Based on your comment, we used mini EDHS 2019 report to indicate the current delivery service coverage but this report did not show the coverage of delivery services based on regions and also did not include maternal mortality rate. Thus we used both EDHS 2016, and mini 2019 reports.**

3) Page 5, paragraph 3 states transport challenges citing more than 8 years old data. Please update it. The extent problem regarding transport access is not the same as the problem before 2012. Over the last few years, the GoE distributed ambulances to districts to expand access to emergency transport though efficient use and universal access is still a challenge.

**Authors’ response: Even if there is improvement, transport access is still a major problem, and still due to lack of modern transport, people use a locally made stretcher which is supported by the result of this study which revealed 72.24% of the respondents reported they were unlikely to get transportation access or walk to long distance in the study area.**

Methods

1. The study utilized pregnant women registered in the selected Kebeles as sampling frame and randomly select respondents from that frame. However, it is not well described how pregnant women were registered in the Kebele? How complete the registration was? And how the women or her household was located for interview? It is known the Ethiopian community health information system/family folder or pregnant women registration by HEWs/WDAs are incomplete. As such, this would induce selection bias due to incomplete sampling frame, potentially excluding eligible respondents

**Authors’ response: In each kebele there are assigned HEWs and these HEWs provide community visits at house hold level at least 2 times	a week and one of their responsibility is to investigate whether pregnant woman/s is present in that hose hold or not , and then register them. The registration includes the gestational age, EDD, Name, Phone number (if available), the exact location and others. Using this registration list of pregnant women as a frame work, we carried out simple random sampling	technique, and interview was done at the household for those randomly selected pregnant women. No selection bias because it was random. To conduct community based study, no other best option to get pregnant women rather than the method that we used.**

2. The analysis section doesn’t mention of account for clustering or not.

**Authors’ response: This study was conducted in Gamo Gofa zone, and districts in this zone were considered as clusters because there is a homogeneous feature between districts. Due to the number of pregnant women estimation in each district, and our sample size, Kebma district was selected randomly. Then we considered that there is a heterogeneous feature with in a cluster, thus from 43 kebeles in Kemba district we took 25% of them (11 kebeles) randomly. Finally proportional allocation was done in each selected stratum (kebele)**

3. The outcome variable originally collected as ordinal but later reduced to binary for logistic regression analysis. I think this technique wastes information and may dilute the statistical power. Why you don’t use ordinal regression?

**Authors’ response: Yes, we agree with your comment, however many of the literatures that we cited provide binary logistic regression, and to make a discussion with these literatures, we have conducted binary logistic regression.**

4. Ethical clearance: Not clear whether written or verbal consent was obtained. And was ethical clearance or support letter this study sought from district health office?

**Authors’ response: The consent that obtained from each participant was verbal consent and before we start data collection support letter was obtained from all respective bodies including the Kemba district health office.**

Results

1) Figure 2 and 3 are already narrated in the text. No need to present same information with different format.

**Authors’ response: I accept your comment, and both Figures are included in the supportive information**

2) Overall, the result section is presented multiple subheadings without adequate description of the findings. For instance, the Table 4 is narrated in the discussion section. I would bring the results described in the discussion section (narrations with figures with odds ratios) to here.

**Authors’ response: Revision is made based on your comment**

3) Contrary to other studies, maternal education is not showing effect on the women’s intention to use MWHs. I think it can be related with the model fit or uncontrolled confounding. For instance, being government employee is associated with intention to use MWHs. This could be due to interaction or confounding with education. Please review it again.

**Authors’ response:** **Model fitness was assured using Hosmer-Lemeshow goodness of fit and also the mode was built via forward stepwise method and we used Variance Inflation Factor (VIF) to check multicollinearity between independent variables, but no more than 15 % change of the B coefficient.**

Discussion

1. Summary the main findings in the first paragraph and dedicate subsequent paragraphs for discussing intention to use and major determinants. Don’t repeat results here (as described above please move the descriptions regarding Table 4 to results section

**Authors’ response: Revision is made based on your comment**

2. There are potential biases in this study including selection bias described above, interviewer bias, and social desirability bias that are not mentioned in the discussion section.

**Authors’ response: Revision is made based on your comment and selection bias, interviewer bias, and social desirability bias are mentioned in the discussion section as the following.**

To avoid selection bias, we used simple random sampling technique to select the study participants from the list of pregnant women in the health extension workers registration book and the data were collected in community at home. During the interview, social desirability bias might be committed, but to reduce it only volunteer participants involved, and their names were kept anonymous, a brief overview of the study was provided, we used experienced interviewers, and the interview was carried out using a one-on-one strategy. To avoid interviewer bias, we instruct our interviewers to read each question accurately to the respondents to answer based on their best understanding of the question, do not interpret the question for the respondents, and offer to say again the question precisely as it appears. Generally, we educate our data collectors to avoid any adjustment, explanation, addition, subtraction, suggestion or change in verbal variation during the interview process.

3. Conclusion is simply repeating the main finding. Please rewrite to reflect the relevance of the findings to the program, implications to future research, or your concluding comments/take-home messages

**Authors’ response: Revision is made based on your comment and it is corrected as the following**

Generally, the intention to use maternity waiting home among pregnant women is less than half, indicated that it is not satisfactory which leads inaccessibility of obstetric cares and it will provoke pregnant women to give birth at home without skill birth attendants that may end up with development  of different complications including maternal and neonatal deaths. And intention to use MWH is significantly associated with occupation of respondents, history of previous childbirth, past experiences of MWH utilization, direct and indirect subjective norm, and perceived behavioral control. These findings call for the intervention of the community to increase the intension of pregnant women towards MWH utilization and we authors recommend that:

4. Some of the recommendations are not grounded from the study findings. For instance, bullet # 2 on page 16

**Authors’ response: we recommend bullet # 2 based on the findings of this study which revealed 81.82% of the respondents reported that it's difficult to get enough food in MWH during service utilization; and 72.24% of them reported they were unlikely to get transportation or walk to long distance.**

5. Language

Finally, there are multiple grammatical errors that needs to edited by someone who has experiences in academic edition. For instance, page 4, paragraph 1 that reads..."one of the three..." not clear what are these; page 5, last paragraph first sentence talks delivery by SBA, but second sentence talks about home delivery. These are not parallel. The third sentence, "...within three.." need to be deleted; page 7, last paragraph first sentence,"... population proportion of 57.3%..." replace by population proportion of intention to use MWHs, 57.3%;

**Authors’ response: Based on your comment, language edition is made.**
