# Supplementary material for: Intention to use maternity waiting home and associated factors among pregnant women in Gamo Gofa zone, Southern Ethiopia, 2019
Source: PLoS One. 2021 May 13;16(5):e0251196. doi: 10.1371/journal.pone.0251196 (PMC8118329; doi:10.1371/journal.pone.0251196)
Supplement: Attachment — Submitted filename: Response to Reviewers.docx [file pone.0251196.s012.docx]

PONE-D-20-31704

Intention to use maternity waiting home and associated factors among pregnant women in Gamo Gofa zone, Southern Ethiopia, 2019

Dear Frank T. Spradley

Academic Editor of PLOS ONE journal

We Authors would like to thank you for your constructive comments, and suggestions. In accordance with the reviewer’s comments, we made a revision. Therefore, we submitted a revised version of the manuscript that addresses the points raised during the review process.

Response to reviewers’ comments

Dear Gizachew Tadele Tiruneh, we Authors would like to thank you for your helpful comments for the second time and based on the comments had given we made revision.

## Reviewer # 2: Major comments 1. 11 paragraphs introduction is too much for a paper. Reduce to 5-6 paragraphs. Authors’ response: The introduction part of the manuscript is reduced to 5 paragraphs based on your comment. 2. Though you described how HEWs are updating pregnant women registration that is not practical. You cannot be sure the registration is complete. In this case, I expect you to do ad hoc list of pregnant women with the help of the HEWs and WDAs before data collection. As such, this would induce selection bias due to incomplete sampling frame. You need to acknowledge this in the limitation section. Besides, you deployed HEWs as data collectors. This would also induce social desirability bias. Acknowledge this also as a limitation and discuss the implications of these biases on the observed results. Authors’ response: It was very difficult to conduct household survey to identify the number of pregnant women in that dispersed community. That is why we used the list of pregnant women from HEWs with its limitation (it may not include new pregnant women) to get the sampling frame. And acknowledgment is done for this limitation as your comment. The rationale of using HEWs as data collectors is that the sampling technique is simple random sampling and the secreted women in each kebele were very far apart to each other, and if we allocate another data collectors, it may bring false data. But the HEWs know the exact location of each pregnant woman and it is easy to collect the data which eliminate the probability of false recording. As you stated, using HEWs as data collectors might impose social desirability (response) bias and it might increase false positive response. However, the observed results did not show the presence of a significant response bias because the magnitude of intention to use MWH is less than 50% which is low, and the result of a favorable attitude, a favorable subjective norm and perceived behavioral control of the direct TPB components were below 50% and also the indirect attitude, indirect subjective norm, and indirect perceived behavioral control of the respondents were about 50% and less than it and about 50% of the participants agreed that HEWs thinks that they should stay in MWH and about 50% the respondents also reported that HEWs’ approval for their stay in MWH was much important. Similarly, more than 80% of the respondents reported that it's unlikely to get enough food in MWH. So based on these and other findings, we believed that the episode of social desirability bias was very minimal/unlikely. Even if it is, we acknowledged it in the limitation part based on your comment.

3. You used statistical criteria for selection of variables to include into the multivariate model. Why you did not use your theoretical framework? And you kept silent about the negative findings about the association between intention to use MWHs and the independent variables maternal education and attitude which is contrary to other studies. In such a behavioral study, it is good to base on conceptual frameworks to select variables. As such, you should force to retain “attitude” and “maternal education” in the model. It can also be related with the model fit or uncontrolled confounding. For instance, being government employee is associated with intention to use MWHs. This could be due to interaction or confounding with education. Besides, gravidity (previous history of childbirth) and past experience of MWHs would be correlated. Please review your analysis again, add possible interactions, force retain attitude and maternal education in the model. And if still, there is no significant associations with your outcome variable, please discuss the negative findings as well. These are important independent variables of interest.
**Authors’ response:** To select variables for the final model we considered two methods: 1) the statistical method that is P-value of less than 0.25 rather than 0.05 to increase the number of variables, which is one of the recommended methods for variable selection. 2) If the variable is significantly associated with intention to use MWH in other researches and if that variable is very important predictor of the outcome, we considered it to be included in the finale model. We prefer to use the statistical method of variable selection rather than the theoretical framework because the final model result is very disrupted if we include a variable which is extremely insignificant in the bivariate analysis and also which is not significant in other research findings. But fortunately, the variables maternal education and both direct and indirect attitudes of the respondents were included in the final model because the p-value of these variables was less than 0.25 and then became insignificant in the multivariate analysis. For your evidence, in the bivariate analysis age of the respondents, educational level, occupation, previous childbirth history, experience of MWH utilization, reason for past utilization, and duration of stay at MWH, direct and indirect attitude, direct and indirect subjective norm, and direct and indirect perceived behavioral control were associated with intention to use MWH with P-value of less than 0.25. To assess the interaction between maternal educational level and government employee, and between previous childbirth history and past experience of MWH utilization, we created new variables and these two new variables were included in the model but were remain insignificant with p-value of greater than 0.05. Based on your comment the negative findings also discussed.
4. Discussion section is still mere repetition of results (of course, you compared with previous literature). You did not summary the main findings, and interpret and discuss implications of your main findings. For instance, in your conclusion you mentioned subjective norm and perceived behavioral control as predictors to intention to use MWHs. You should interpret what subjective norms and perceived behavioral control mean and state as take home messages to readers. Think of it. Subjective norm and perceived behavioral control are still jargons. Can it be interpreted as low subjective norm or high community disapproval, low perceived benefits, and low self-efficacy? If so, you may summarize the main findings as such “The intention to use MWHs is low. Community disapproval, low self-efficacy, and maternal employment…are predictors to use MWHs” And discuss each main finding dedicating a paragraph, of course, without repeating the results.

**Authors’ response:** Discussion part of the manuscript is revised based on your comments. In this study subjective norm of pregnant women to use MWHs is measured as favorable or unfavorable based on the mean value which indicated that pregnant women with unfavorable subjective norm have low subjective norm or high community disapproval. And the concept of self-efficacy is used as perceived behavioral control, which means the perception of the ease or difficulty of pregnant women to use MWHs and unfavorable perceived behavioral control indicates low self-efficacy.

5. Language: Still there are multiple grammatical errors that need to edit by someone who has experiences in academic edition.

**Authors’ response**: Basic edition is carried out in the whole document.
**Minor comments**
• Abstract: correct the # of respondents to 605: **It is corrected from 621 to 605**
• Use of consistent decimal point across the text. **It is checked.**
• You did not narrate the prevalence of intention to use MWHs in the results section. Please narrate it. It is already narrated in the first 4 lines of the result section of the manuscript without subheadings but now subheading “**prevalence of intention to use MHHs**” is included.
